# Biochemical and cellular characterization of transcription factors binding to the hyperconserved core promoter-associated M4 motif

**DOI:** 10.1186/s12864-016-3033-3

**Published:** 2016-08-30

**Authors:** Ngo Tat Trung, Elisabeth Kremmer, Gerhard Mittler

**Affiliations:** 1 Department of Cellular and Molecular Immunology, Proteomics Core Facility, Max-Planck-Institute of Immunobiology and Epigenetics, D-79108 Freiburg, Germany; 2 Faculty of Biology, Albert-Ludwigs-University Freiburg, Schänzlestrasse 1, D-79104 Freiburg, Germany; 3 Present address: Tran Hung Dao University Hospital (Benh Vien TWQD 108), No 1, Tran Hung Dao Str, Hai Ba Trung Dist, Hanoi Vietnam; 4 Institute of Molecular Immunology, Helmholtz Center Munich, German Research Center for Environmental Health (GmbH), D-81377 München, Germany; 5 Present address: Ludwig-Maximilians-University Munich, Biocenter, Humanbiologie und Bioimaging, D-82152 Martinsried, Germany; 6 BIOSS, Center for Biological Signalling Studies, University of Freiburg, Schänzlestr. 18, D-79104 Freiburg, Germany

## Abstract

**Background:**

The motif ACTAYRNNNCCCR (Y being C or T, R being A or G, and N any nucleotide), called M4, was discovered as a putative cis-regulatory element, present 520 times in human promoter regions. Of these, 317 (61 %) are conserved within promoter sequences of four related organisms: human, mouse, rat, and dog. Recent genome-wide studies have described M4 as a transcription factor (TF) binding site for THAP11 that does often overlap with SBS (STAF Binding Site) a second core-promoter associated TF binding module, which associates with the TFs STAF/ZNF143 and RBP-J. Human M4-promoter genes show enhanced expression in cells of hematopoietic origin, especially in B lymphoblasts and peripheral blood B and T cells. Apart from RBP-J that is well known to recruit ICN1 (the intracellular transcriptional mediator of activated Notch1), the functional role of the hyperconserved M4 cis-element in the context of transcriptional regulation of M4-genes in lymphoid cells remains poorly defined.

**Results:**

Here, we present a quantitative proteomic investigation of the M4 motif TF binding landscape in lymphoid cell lines that is further validated by ChIP experiments and functional assays. Our data strongly suggest that THAP11 and Ikaros interact directly, while NFKB1 (NF-kappa B p50) and HCF-1 are binding indirectly to M4-promoters in vitro and in living cells.

Further analysis reveals that M4 is a bipartite composite cis-element, which is recognized by THAP11 via binding to the ACTAYR sequence module, thereby promoting ternary complex formation with HCF-1. Similarly, Ikaros binds to the CCCR module of the M4 motif and this interaction is crucial for recruiting NFKB1 to M4 harboring genes. Transient reporter assays in HEK293 and loss-of-function experiments in Molt4 T cells unequivocally demonstrate that binding of Ikaros and/or THAP11 to M4 bearing promoters is functionally important and therefore biologically relevant. Accordingly, this study validates our SILAC-based DNA protein interaction screening methodology as a valuable surrogate for a bona fide reverse ChIP technology.

**Conclusions:**

The M4 motif (ACTAYRNNNCCCR) is a functional regulatory bipartite cis-element, which engages a THAP11/HCF-1 complex via binding to the ACTAYR module, while the CCCRRNRNRC subsequence part constitutes a binding platform for Ikaros and NFKB1.

**Electronic supplementary material:**

The online version of this article (doi:10.1186/s12864-016-3033-3) contains supplementary material, which is available to authorized users.

## Background

Of the roughly 20,000 protein-coding (Genecode 25/Ensembl 85) genes [1], there are approximately 8000 to 10,000 genes expressed at a significant level in a given cell type. The gene regulatory code consists of the DNA sequence-dependent binding specificities of a prominent class of DNA binding proteins. These so called transcription factors (TFs) are able to read the regulatory information and transmit the information encoded in primary DNA sequence to the transcriptional machinery, thereby regulating the synthesis of RNA transcripts [2]. Differential tissue-specific gene expression is an essential component of gene regulation in humans, which restricts the expression of certain genes to given stages of development. Importantly, the erroneous reading of the gene regulatory code is often accompanied by the formation of tumors or the manifestation of other diseases associated with developmental disorders. Therefore, a detailed understanding of the spatial and temporal gene expression patterns is of fundamental importance for understanding the complexity of the human genome. This is by far not a trivial task because the cis-regulatory elements are usually ambiguous and context-dependent and can function both in the immediate vicinity of genes and as enhancer elements [3] thousands of DNA base pairs away from their transcriptional start site.

Since the cis-regulatory elements and associated transcription factors are believed to be responsible for maintaining the conserved gene expression patterns between related organisms, the elucidation of the gene regulatory code brings back into focus a simple question: “Which transcription factors (TFs) do bind to a cis-regulatory element in a given cell type?”

Traditionally, TF binding to a putative cis-element could be identified through the combination of classical biochemical fractionations coupled to Eletrophoretic Mobility Shift Assay (EMSA) and amino acid sequencing of the purified transcription factors.

In order to significantly improve the traditional approach, it is essential to link the isolation and enrichment of the TF directly to the detection of its sequence specificity and its identification. Recently, we [4] and others [5] have developed scalable methodologies [6] that are fulfilling this task. Our SILAC-based DNA protein interaction screen [4] makes use of the fact that one can introduce mutational changes in the DNA sequence to destroy the function of a cis-element thereby preventing the binding of its regulatory TFs. Hence, a direct quantitative proteomic comparison of the protein binding pattern between the natural and the mutated DNA sequence via nanoLC-MS will provide the identity of the TFs that have the potential to interact with the regulatory element in vivo.

Here we apply this technology to investigate an evolutionary hyperconserved promoter-associated cis-element, termed M4 that until now has been poorly studied. Briefly, a modern bioinformatic whole genome alignment comparison of mammalian genomes (often coined “phylogenetic footprinting”) led to the discovery of several novel, evolutionarily conserved cis-elements, whose function was in most cases poorly characterized [7, 8]. Among them the best conserved novel element found was the M4 motif with the DNA consensus sequence “RRACTACANNTCCCRRNRNRC.” This was partially described as a binding site for the non-lymphoid transcription factors THAP11, STAF/ZFN143 and RBP-J [9, 10]. As suggested by the fact that genes harboring M4 motifs exhibit increased expression levels in lymphoid and some leukemia or lymphoma cells [7], it implied a putative role of M4 motif in the lymphoid system or in hematopoietic malignancies.

In the present study, by using SILAC-based quantitative proteomics, we show that the lymphocyte specific Ikaros family proteins (Ikaros, Aiolos, and Helios), the NF-kappa B proteins NFKB1 and NFKB2 (NF-kappa B p50 and NF-kappa B p52), the pluripotency factor Ronin/THAP11 and host cell factor-1 (HCF-1) are able to bind specifically, either directly or indirectly, to the M4 motif in vitro. Importantly, the TF M4-DNA interactions of Ikaros, NFKB1 (NF-kappa B p50), THAP11 and HCF-1 are confirmed by ChIP assays in living cells, thus providing evidence that the SILAC-based DNA protein interaction screen can work as a surrogate for a bona fide reverse ChIP technology. Notably, our data also suggest that the conserved M4 motif is a composite cis-element containing two TF binding modules, which can operate independently from each other. We provide strong evidence that THAP11 interacts directly with the ACTAYR part of M4 thereby engaging in a ternary complex with HCF-1, whereas Ikaros binds to the CCCRRNRNRC sequence of M4. The latter interaction is essential for recruiting NFKB1 (NF-kappa B p50) to the M4 motif. Transient reporter assays performed in HEK 293 cells and sh-RNA mediated loss-of-function assays in Molt4 T cells unequivocally demonstrate binding of Ikaros and THAP11 to genes containing M4-promoters as functional and therefore biologically relevant. Our current model, utilizing transient reporter assays and THAP11 shRNA knockdown in Molt4 T cells, mainly suggests an activator function of THAP11/HCF-1 for M4-genes in these cells. Unlike THAP11, the apparent role of Ikaros is to modulate the transcriptional output of endogenous M4-harboring genes by either repressing or activating them.

## Results

### EMSA assay reveals presence of M4 binding factors in lymphocyte nuclear extracts

**Fig. 1:**
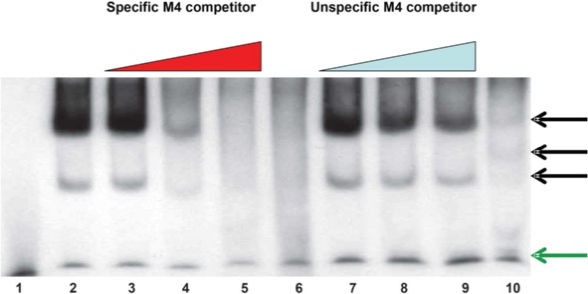
EMSA assay to uncover lymphocyte-specific transcription factors interacting with M4 motif bearing DNA. Radioactive γ-ATP labeled M4 oligo was incubated either with Raji burkitt’s lymphoma nuclear extracts (lane 2, 3, 4, 5, 7, 8, 9), or Hela nuclear extract (lane 10). EMSA complexes formed in assays using Raji burkitt’s lymphoma nuclear extract (lane 2, 3, 4, 5) are virtually absent in Hela nuclear extract (*black arrow bar*). Those band shifts were significantly faded away when an increasing amount of cold M4 motif was added (lane 2,3,4,5) but still preserved (lane 7, 8, 9) if the cold M4 motif containing oligo was replaced by an M4 scrambled counterpart. Lanes 1, 6 are control assays using M4 oligo in the absence of nuclear extracts

Although several studies have shown the binding of some TFs to the M4 motif [9–11], none reported M4 as the binding site for a heamatopoietic TF regardless of the fact that genes harboring the M4 motif exhibit higher expression levels in many cell lines derived from the immune system [7]. EMSA was performed using a radioactively labeled double-stranded oligodeoxynucleotide bearing the M4 motif that was incubated with either Raji burkitt’s lymphoma or Hela nuclear extracts followed by electrophoretic separation on a native polyacrylamide gel in order to reveal potential M4 binders that are specifically expressed in hematopoietic cells. The band shift data from Fig. 1 show the presence of lymphocyte-specific (Raji nuclear extract) M4 binding factors that are hardly expressed in cervical cancer HeLa cells (lane 10). Importantly, the Raji lymphoma specific band shifts are still formed even in the presence of a hundred-fold excess of an unlabeled competitor oligonucleotide bearing a sequence unrelated to M4 (lane 7, 8, 9). Conversely, the formation of band shifts is completely abolished if a hundred-fold excess of non-radioactive M4 oligonucleotide is added (lane 2, 3, 4, 5). This suggests that Raji burkitt’s lymphoma cells express proteins that can selectively interact with M4 motif in vitro.

### MS-based proteomic screening to identify lymphocyte-specific M4 interacting factors

**Fig. 2:**
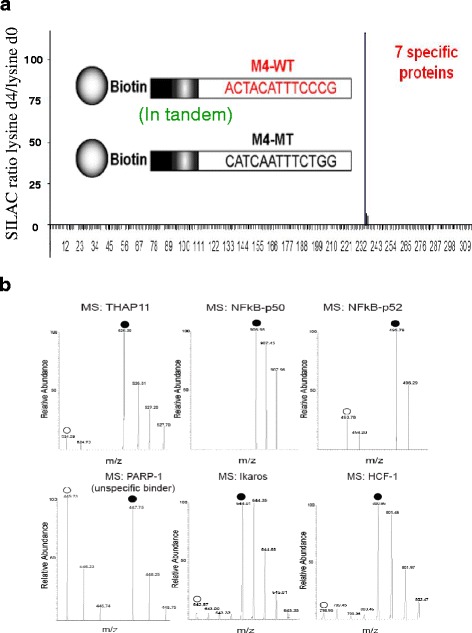
SILAC-based DNA protein interaction sreen reveals M4 binding candidates. **a** Distribution of SILAC ratio against quantified proteins identified in tandem M4 DNA affinity chromatography experiments using Raji burkitt’s lymphoma nuclear extracts: The X-axis present the random code of quantified proteins originating from the mass spectrometric screening of M4 DNA affinity chromatography experiments; the (Y axis) present SILAC ratios of individually quantified proteins; **b** Mass spectra of selected M4 interacting partners’ derived peptides: mass spectra of individual peptides comprising both light isotope (*white circle*) and heavy peaks (*black circle*). Those derived from specific M4 binders were specified by higher intensity of heavy isotope encoded peaks. In contrast, the ones descending from unspecific binders are presented by equal intensity of both light and heavy isotope encoded peaks (in this case PARP-1)

**Fig. 3:**
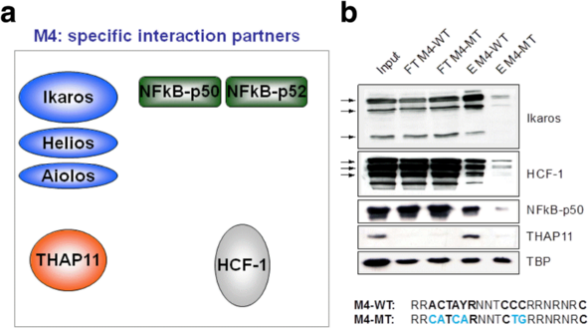
Immunoblot analysis to confirm the specific binding of Ikaros, HCF-1, NFKB1 (NF kappa B p50) and THAP11 to the mono M4 motif-containing DNA affinity column. **a** prominent putative M4 binding candidate factors. **b** DNA affinity chromatography: M4 WT and control mono M4 MT were immobilized as ligand, Raji burkitt’s lymphoma nuclear extract was used as input. Protein-DNA complexes from two columns were eluted, resolved by SDS-PAGE separately for Western blotting analysis. Specific antibodies were used to detect the retention of suspected M4 binding candidates. The unspecific DNA binding factor TPB was adopted as loading control: (Abbreviation: FT-WT M4/flow-through M4 wild type, FT-M4-MT/ flow-through M4 mutant, E M4-WT/ eluate M4 wild type, E M4 –MT/ Eluate M4 mutant)

To identify M4 interacting factors, Raji burkitt’s lymphoma cells were metabolically encoded with either “heavy” or “light” (non-radioactive) isotopic variants of lysine (^2^H_4_ lysine/K4 or ^1^H_4_ lysine/K0). The corresponding nuclear extracts (NEs) were prepared and used as an input for subsequent SILAC-based DNA affinity pull-down screening [4, 12, 13] comparing the protein binding profile of M4 wild-type (WT) versus M4 mutant baits (Fig. 2). Following trypsin digestion and nanoLC-MS 314 out of more than 1000 identified proteins were quantified by the MSQuant software [14] utilizing lysine-containing tryptic peptides. Most of the isotopic variants of quantified peptides were present in almost equal amounts (indicated by approximate one-to-one SILAC ratios-Fig. 2a and b). This unequivocally demonstrated that most of the quantified proteins do bind unspecifically to either M4 WT or M4 MT columns. However, there were seven candidate factors (Table 1, Fig. 2a and b), exhibiting SILAC ratios greater than two that were termed M4-binding candidates (Fig. 3a).

**Table 1:** Positive tandem M4 binding candidate proteins in Raji burkitt’s lymphoma cell line

M4 motif binding candidate proteins	Uniprot identifier	Mascot score	Peptide No	Peptide No used for quantification	SILAC ratio
Ikaros/IKZF1	Q13422	658	16	5	>100
Aiolos/IKZF3	Q9UKT9	27	1	1	>100
Helios/IKZF2	Q9UKS7	43	2	1	>100
NFKB1 (NF-kappa B p105/50)	P19838	232	8	2	19
NFKB2 (NF-kappa B p100/52	Q00653	218	6	2	31.8
HCFC1	P51610	381	9	5	19.0
THAP11	Q96EK4	37	3	1	11.0

### Immunoblot analysis confirms the in vitro interaction of M4 binding candidates with M4

The M4 sequence of the oligodesoxynucleotide baits used in the proteomic screening experiment were derived from the DFFA gene (DNA fragmentation factor alpha) promoter, which contains two copies of the M4 motif in close vicinity (Fig. 2 “in tandem”); however, most human M4 bearing genes contain only one copy of the M4 motif in their promoter [8]. To exclude the possibility that a duplicated M4 motif could induce some avidity binding effects in respect to putative M4 interacting factors, the DNA affinity chromatography was repeated using columns that harbor only one copy of either WT or mutant M4 motif. Separate eluates from WT and MT DNA columns were resolved by SDS-PAGE and subjected to immunoblot analysis for validating the putative M4-binding candidates. As shown in Fig. 3, antibodies directed against Ikaros, host cell factor 1 (HCF-1), NFKB1 (NF-kappa B p50), and THAP11 confirmed the specific binding of these proteins to the WT M4 motif (Fig. 3b).

### M4-binding proteins interact with promoter regions containing the M4 motif in vivo

To demonstrate the potential biological function of M4 motif binding candidates, classical formaldehyde crosslinking chromatin immunoprecipitation (ChIP) assays were performed, followed by qRT-PCR of bound DNA. In this assay, the three M4 bearing promoters of the genes Skp2, CDC25A, and G9A were selected for two reasons: First, gene ontology analysis of M4-genes reveal that genes involved in mitotic cell cycle regulation and chromatin function are statistically overrepresented amongst M4 bearing genes (data not shown). Second, expression levels of the three aformentioned genes were significantly affected upon ectopic over-expression of the dominant negative Ik6 isoform [15] of Ikaros in Raji burkitt’s lymphoma cells as well as Molt4 T-cells (data not shown). Initial attempts to establish ChIP conditions for Ikaros in Raji burkitt’s lymphoma cells employing antibodies directed against the Ikaros C-terminus were difficult and could be ascribed to the fact that Raji burkitt’s lymphoma cells exhibit an abundant expression of small Ikaros isoforms (data not shown), which are not able to bind DNA directly but can multimerize with IK1 and Ik2 (DNA binding) isoforms. Such higher-order complexes cannot be efficiently recovered by the standard ChIP protocol, which works under stringent denaturing conditions strongly favouring the recovery of DNA sequences that are directly crosslinked to the immunoprecipitated protein.

To circumvent this, we decided to focus on T-lymphoma Molt4 cells because these cells do not express the small Ikaros isoforms (data not shown). Furthermore, to standardize our IP conditions, C-terminally HA-tagged Ikaros that is known to recognize canonical Ikaros target genes [16] was constructed and retrovirally transduced into Molt4 cells (Ikaros-HA transduced cells are termed Molt-IK-HA hereafter). A similar procedure was adopted for the generation of Molt4-THAP11-HA cells, which ectopically expresses C-terminally HA-tagged THAP11 protein. Importantly, like its endogenous counterpart, THAP-11-HA demonstrates robust M4-specific binding in a DNA affinity pulldown assay (Additional file 1: Figure S1).

**Fig. 4:**
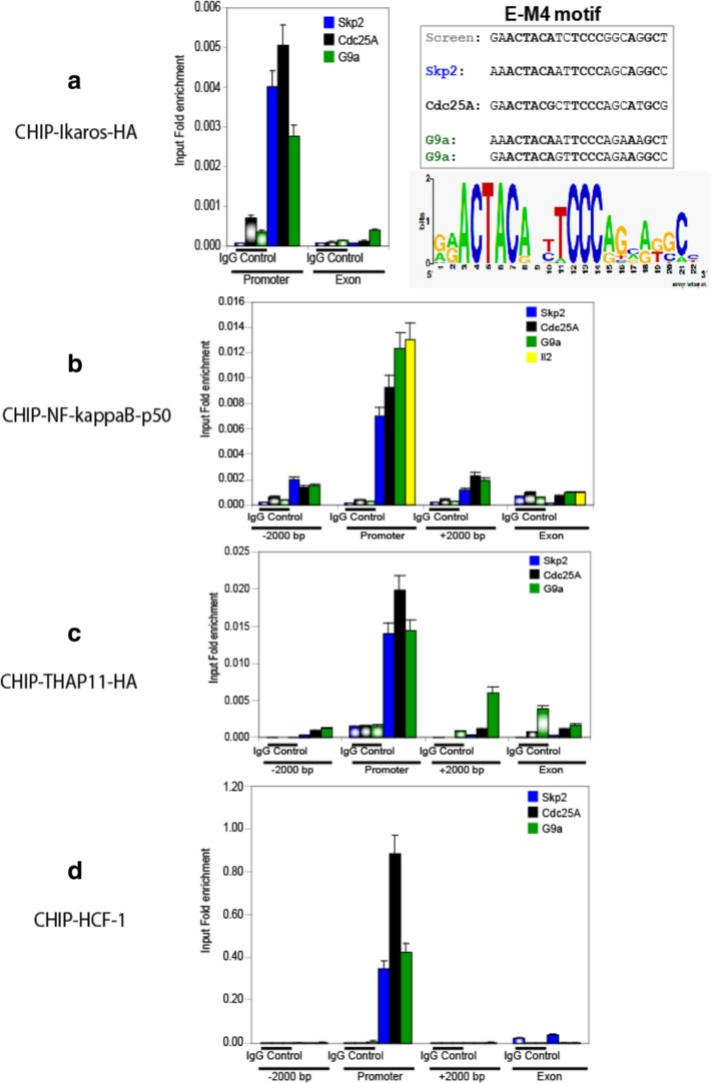
Chromatin Immunoprecipitation (ChIP) analysis of Ikaros, NFKB1 (NF-kappa B p50), THAP11, and HCF-1 binding to M4-bearing promoters of the genes Skp2, CDC25A, and G9a. Formaldehyde crosslinked chromatin of Molt-IK-HA or Molt4-THAP11-HA cells were sheared by ultrasonication into an average length of 300 to 1000 bps to be subjected to immuno-precipitation assays using anti-NF-kappa B p50, anti-HCF-1, anti-HA/Ikaros, anti-HA/THAP11, or preimmune IgG sera (control). The TF-associated chromatin were analyzed via quantitative real time PCR (qRT-PCR) using primer sets flanking the suspected TF binding site and background binding control regions: Promoter (M4 motif), Exon (exon control), ±2000 bp (control regions downstream and upstream from the corresponding M4 cis-element). Enrichment of TF-bound DNA amount were normalized to corresponding amount in input. ChIP results (average of three technical replicates) for Ikaros (**a**), NFKB1 (NF-kappa B p50) (**b**), THAP11 (**c**) and HCF-1 (**d**) are shown

The two HA-tagged transgenic Molt4 T-cell lines were later used for either Ikaros or THAP11 ChIP assay using a high affinity rat-anti-HA antibody (3 F10). As seen in Fig. 4a, M4 motif-bearing DNA was specifically co-precipitated by anti-Ikaros/HA IP (compared with IgG control serum), demonstrating specificity of the IP protocol. Strikingly, binding of Ikaros to chromatin was selectively enriched on DNA fragments containing the M4 motif in all three promoters examined (Skp2, CDC25A, and G9a). Therefore, it can be concluded that Ikaros interacts with the M4 cis-element in living cells. Recapitulating the same experimental strategy, we were able to show the specific and selective enrichments of NFKB1 (NF-kappa B p50) (Fig. 4b), THAP11-HA (Fig. 4c) and HCF-1 (Fig. 4d) on the promoters of the M4 bearing genes Skp2, CDC25A, and G9a.

Intriguingly, ChIP enrichment of NFKB1 (NF-kappa B p50) bound interleukin-2 promoter DNA, which represents a well established NF-kappa B target gene harboring a classical palindromic kappa B binding site [17, 18], was comparable to the enrichment of NFKB1 (NF-kappa B p50) bound M4-promoter DNA (Fig 4b). This suggested that the affinity of NFKB1 (NF-kappa B p50) towards M4-bearing promoters was not substantially different compared to a well studied classical NF-kappa B target gene.

### M4 motif is a composite element, comprising two distinct non-overlapping binding sites for both THAP11 and Ikaros

According to our studies the M4 motif consists of two highly conserved parts. While the “upstream core” RRACTAYR is similiar to the Ronin (murine THAP11 orthologue) binding site, the “downstream core” (TCCCRRNR) is similar to the previously described murine Ikaros binding site consensus (YCTCCCARR) [19]. This raises the question if the M4 motif is indeed a composite element that can be partitioned into two sub-consensus sequences. The latter can be best answered by investigating if the suspected direct sequence-specific interaction partners of the M4 cis-element, THAP11 and Ikaros, are binding synergistically or independently to M4.

**Fig. 5:**
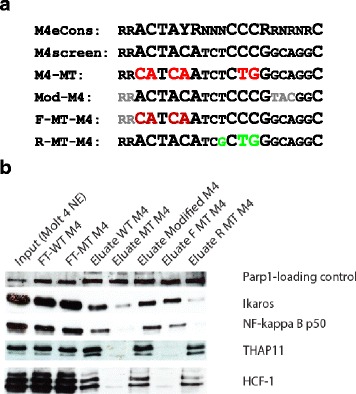
The M4 motif is a bipartite composite element, consisting of two independent binding site modules for THAP11 and *Ikaros.* **a** DNA ligands used in affinity chromatography: mono-M4 wild-type (WT), mono-M4 mutant (MT), Mod-M4 (the very flanking nucleotides of the M4 motif are modified), F-MT-M4 (the core” RRACTAYR of M4 motif was modified) and R-MT-M4 (the “downstream core” TCCCRRNR of M4 motif were modified). **b** Immunblot analysis of DNA affinity chromatography experiments using five DNA ligands: Input (Molt4 nuclear extract) and eluates from three columns were resolved by SDS-PAGE in parallel and subjected to Western blotting. Specific antibodies detected the retention of suspected M4 binding factors

Therefore, we hypothesized that the “downstream core” (TCCCRRNR) will be sufficient to recruit Ikaros, whereas the “upstream core” RRACTAYR would be indispensable for engaging other M4 binders namely THAP11. To verify this, oligo-pulldown assays were conducted using three modified M4 motif-bearing DNA sequences as a ligand: Mod-M4 (the very flanking nucleotides of the M4 motif were mutated), F-MT-M4 (for which the “upstream core” RRACTAYR of M4 motif was mutated), and R-MT-M4 (the “downstream core” TCCCRRNR of M4 motif was mutated). As can be seen in Fig. 5, the “flanking” mutations did not interfer with the binding of either M4 binding factors to the M4 motif. In contrast, the mutation of the M4 “upstream core” RRACTAYR was sufficient to simultaneously abrogate the binding of THAP11 and HCF-1 to M4 motif bearing DNA, while still preserving the interaction of both Ikaros and NFKB1 (NF-kappaB p50) with the M4 motif. Consequently, mutation of the “downstream” part of the M4-motif abolished recruitment of Ikaros and NFKB1 (NF-kappaB p50) to M4 DNA, but did virtually not alter the binding of THAP11 and HCF-1 to M4. For this reason we conclude from our in vitro binding data that M4 is a composite TF binding module. It consists of two TF binding sites; the “upstream core” RRACTAYR that is essential for recruiting THAP11/HCF-1 to the M4 cis-element and the “downstream core” TCCCRRNR that is required for establishing M4-Ikaros/NF-kappa B interactions.

### NFKB1 (NF-kappa B p50) interaction with M4 is critically dependent on Ikaros

**Fig. 6:**
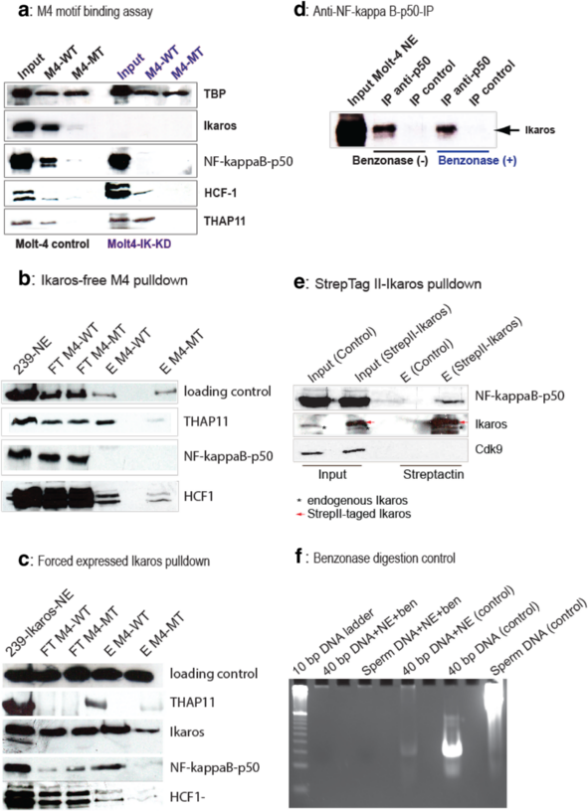
NFKB1 (NF-kappa B p50) interacts with Ikaros and this interaction is essential for recruiting Rel-NF-kappa B family TFs to M4 motif harboring DNA*.* **a** Immunoblot analysis of DNA affinity chromatography experiments using two DNA ligands: mono-M4 WT and mono-M4 MT. Molt4 control or Molt4-Ikaros KD nuclear extracts served as input. In the absence of Ikaros, protein levels of NFKB1 (NF-kappa B p50) were virtually not affected (lane 4 vs 1) but its binding to the M4 column was completely abrogated (lane 5). In contrast, the binding of the two other established M4-binders (THAP11 and HCF1) were robustly maintained (lane 2 and 6). **b** Anti-NF-kappa B p50 (NFKB1) IP (immunoprecipitation) co-precipitated Ikaros in the presence (lane 4,5) and absence (lane 2, 3) of the highly efficient nuclease bezonase. **c** Cross-linked streptactin pulldown experiment demonstrates co-purification of StrepII-tagged Ikaros (see the text) with NFKB1 (NF-kappa B p50) protein (lane 4), whereas the nuclear protein kinase CDK9 that was reported to interact with NFKB1 (NF-kappa B p50) (see text) was not present in the cross-linked complex (compare lane 1, 2 with 3, 4). **d** Benzonase nuclease digestion control assay: the control 40 bp duplex DNA or control salmon sperm DNA were mixed with Molt4 nuclear extracts in the presence or absence of benzonase at 4 °C (5 h to overnight). The whole samples were analyzed by an 8 % (19:1) native TBE-PAGE gel that allows separation of DNA down to the size of 10 bps

The M4 motif is neither a palindromic sequence nor analogous to any known kappa B site [17, 18]. However, the “downstream core” (TCCCRRNR) that mediates NFKB1 association is highly similar to the murine Ikaros binding site. Therefore, one of two scenarios can be envisioned: i) Ikaros and NFKB1 compete in binding to the “downstream core” (TCCCRRNR) of the M4 motif or ii) NFKB1 is recruited to the M4 motif in an Ikaros-dependent manner. To investigate this a stable shRNA Ikaros knock-down Molt4 cell line (named Molt4-IK-KD in the following) was generated by retroviral transduction, in which Ikaros protein expression was almost completely abrogated (Fig. 6a, input Molt4-IK-KD). DNA affinity chromatography experiments using mono-M4 WT and mono-M4 MT beads in combination with nuclear extracts from Molt4 (control), Molt4-IK-KD or cells that do not express Ikaros (human embryonic kidney HEK 293 cells, Fig. 6b) were conducted and subjected to immunblot analysis as described before. Notably, even though protein expression levels of NFKB1 (NF-kappa B p50) were virtually not affected by our highly efficient Ikaros knockdown, its binding to the M4 column was completely abolished. However, upon ectopic expression of Ikaros in HEK293 cells (Fig. 6c), the recruitment of NFKB1 (NF-kappa B p50) to M4 containing DNA was reestablished. In contrast, the binding of the two other M4-binders (THAP11 and HCF1) was not affected either by the absence (Fig. 6a and b, Molt4-IK-KD) or presence (Fig. 6b and c) of Ikaros expression. This implies that Ikaros expression is absolutely crucial for the binding of NFKB1 (NF-kappa B p50) to DNA harboring the M4 sequence in vitro. Nevertheless, it remained to be resolved if NFKB1 (NF-kappa B p50) was recruited to M4 by a more direct interaction with Ikaros. To address this, we have carried out a coimmunoprecipitation assay using anti-NFKB1 (NF-kappa B p50) antibody. The results clearly demonstrate that Ikaros is part of a NFKB1 (NF-kappa B p50) protein complex, which is resistant to efficient (Fig. 6f) DNA and RNA nuclease (benzonase) digestion (Fig. 6d). Of note, the latter indicates that the Ikaros containing NFKB1 complex is not formed or stabilized by random protein nucleic-acid interactions.

To exclude the possibility that Ikaros might unspecifically associate with the anti-NFKB1 (NF-kappa B p50) serum, a reverse immunoprecipitation assay using either anti-Ikaros or anti-HA (Molt-IK-HA cells) antibodies was conducted. However, co-precipitation of NFKB1 (NF-kappa B p50) was not achieved (data not shown). Since both the anti-Ikaros and anti-HA antibody recognize epitopes at the C-terminus of Ikaros, which is known to mediate protein-protein interactions [20], we reasoned that antibody binding to the Ikaros carboxyterminus might disturb the Ikaros-NFKB1 (NF kappa B p50) interaction.

To circumvent this, an aminoterminal tag was fused to the cDNA of human Ikaros (Additional file 1: Figure S2). The tandem-StrepII-tag (StrepOne tag) was selected as it exhibits an extremely fast on-rate during binding to Streptactin beads and chromatography can be performed under very stringent conditions employing formaldehyde crosslinking. In order to pursue this, a Molt4 cell line stably expressing StrepII-tagged Ikaros (Molt4-strep-tagged-Ikaros cells) was created via retroviral transduction. To confirm that the heterologously expressed StrepII-tagged Ikaros protein is functional, ChIP experiments were conducted as described above. The ChIP enrichement values for selected M4-bearing gene promoters (Skp2, CDC25A, and G9a) were almost identical (Additional file 1: Figure S2) to the ones obtained with Ikaros-HA protein (Molt-IK-HA cells, Fig. 4a) suggesting that ectopically expressed StrepII-tagged Ikaros retains its biological function.

As seen in Fig. 6e, the crosslinked Streptactin pulldown experiment reveals a moderate (but highly specific and reproducible) co-purification of NFKB1 (NF-kappa B p50) protein with StrepII-tagged Ikaros, whereas the nuclear protein kinase CDK9 that was reported to directly interact with NFKB1 (NF-kappa B p50) [21] was not present in the crosslinked complex. This demonstrates high specificity of the formaldehyde crosslinking approach (e.g. avoiding “over”-crosslinking). Since the benzonase treatment proved to be very efficient in removing nucleic acids from the input fraction (Fig. 6f), it could be inferred that the Ikaros-NFKB1 (NF-kappa B p50) complex formation most likely involves protein-protein contacts and is not unspecifically mediated by random DNA or RNA. Hence, we conclude (Fig. 6) that a minor fraction of nuclear NFKB1 (NF-kappa B p50) is able to engage with Ikaros and that this interaction is most likely essential for recruiting NFKB1 to M4 motif containing DNA in vitro. Importantly, the data do not rule out the possibility that the Ikaros-NFKB1 (NF-kappa B p50) interaction can be enforced upon binding to M4 DNA, either by an allosteric effect transmitted through a conformational change of Ikaros upon DNA binding or by (sequence-independent) DNA-contacts mediated by the Rel homology domain of NFKB1 (or both).

### Ikaros knock-down does not influence the M4 motif binding of THAP11 and HCF-1 in vivo

**Fig. 7:**
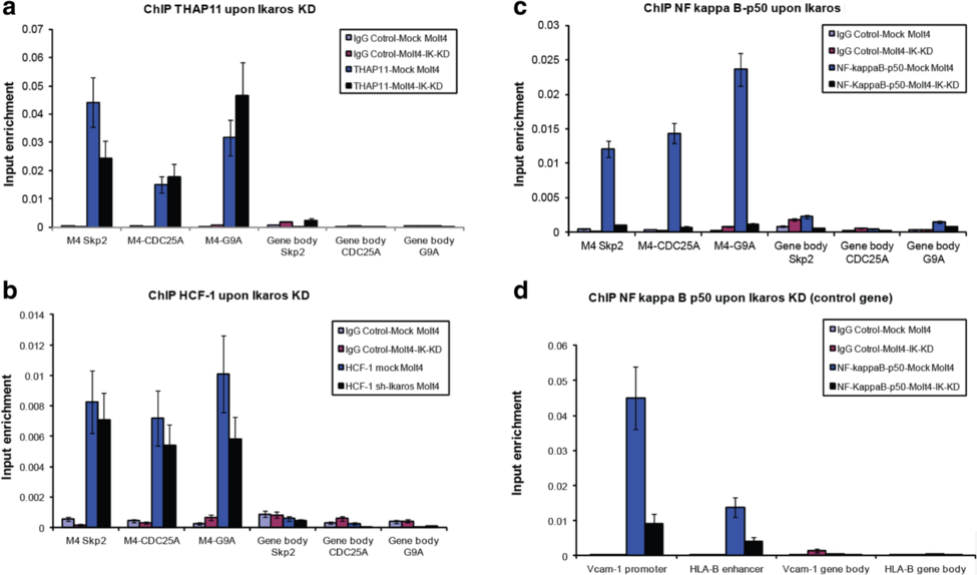
Chromatin Immunoprecipitation (ChIP) analysis of NFKB1 (NF-kappa B p50), THAP11 and HCF-1 interaction with M4 genes in Ikaros knockdown cells*.* Formaldehyde crosslinked chromatin of Molt4 or Molt4-IK-KD cells was sheared by ultrasonication into an average length of 300 to 1000 bps and ultimately subjected to immuno-precipitation assays using **a** anti-THAP11 antibody, **b** anti-NF-kappa B-p50 (NFKB1) antibody, **c** anti HCF-1, or preimmune IgG sera (control). The TF-associated chromatin were analyzed via quantitative real time PCR (qRT-PCR) using primer sets flanking the promoters (M4 motif), and gene body control regions (Gene body) of M4-bearing Skp2, CDC25A, and G9a genes; **d** the known NFKB1 (NF-kappa-B p50) target genes Vcam-1, HLA-B were also included as control. Enrichment of TF-bound DNA amount were normalized to corresponding amount in input. ChIP results (average of three technical replicates) for NFKB1/NF-kappa B p50 (**c**), THAP11 (**a**), and HCF-1 (**b**) were shown

To confirm that the M4 motif does also operate in vivo as a composite element, which assumes that the Ikaros/ NFKB1 (NF-kappa B p50) complex does not influcence the binding of THAP11/HCF-1 to M4-gene promoters, we performed ChIP in Molt4 and Molt-IK-KD cells, respectively (as seen in Fig. 7a and b). In the absence of Ikaros expression (Molt-IK-KD cells), the enrichment of NFKB1 (NF-kappa B p50) at three representative M4 bearing promoters (Skp2, CDC25A, and G9a) was totally abrogated (Fig. 7c), whereas binding of either THAP11 or HCF-1 did not change substantially. Consequently, these data together with the results depicted in Fig. 6 lead us to conclude that the M4 motif also constitutes in vivo a composite cis-element, with distinct and independent THAP11/HCF-1 and Ikaros/ NFKB1 binding modules.

### THAP11 interacts with HCF-1 via its C-terminal domain, which is required for tethering HCF-1 to M4 motif containing DNA

Towards this end we could show that HCF-1 is a bona fide component of M4 motif-binding complexes. However, HCF-1 is not known to be a sequence-specific DNA binding factor but primarily contacts DNA through association with other TFs, such as the Oct1 protein [22] and Ronin-the mouse orthologue of THAP11 [10, 11, 23]. In order to examine if this interaction is also taking place in the context of human proteins, a GST-pulldown assay was carried out using Molt4 nuclear extracts as input. Briefly, the cDNA encoding either full-length or the carboxyterminal part of human THAP11 (encompassing the last 116 amino-acids) were cloned in-frame downstream of a glutathione-S-transferase gene (GST) cassette and expressed in *E. coli*. Subsequently, the purified GST-fusion proteins were used as bait for identifying THAP11-interacting partners from Molt4 nuclear extracts in GST pull-down assays. In this context glutathione-sepharose beads loaded with GST only served as a negative control.

As illustrated in Additional file 1: Figure S3, homogenously purified GST-fusion proteins were obtained (lower panel) that efficiently and specifically captured HCF-1 protein as visualized by immunoblot analysis (upper panel). This result could also be confirmed in an independent proteomic experiment using LC-MS-based protein identification (data not shown). Therefore, we infer that the carboxyterminal domain of THAP11 interacts quite strongly with HCF-1 in human cells, an interaction potentially crucial for tethering HCF-1 to M4 motif-bearing DNA.

### Binding of Ikaros and THAP11 to a M4 motif containing promoter is functional in transient reporter assays

Until now we demonstrated that in lymphocytes, THAP11 and Ikaros independently interact with M4 cis-elements in vitro as well as in living cells. However, it is equally important to assess if M4-binding plays a functional role in regulating gene expression of M4-harboring genes. As a functional readout, we first established a reporter assay system using HEK 293 cells, in which, a set of artificial M4 and M4 mutant-bearing promoters could be studied for their regulatory potential to drive expression of a downstream-conjugated luciferase gene reporter. As discussed above HEK293 cells do not possess any detectable Ikaros expression but ectopic expression is easily accomplished by transient transfection. As already described in Fig. 6b and c, ectopically expressed Ikaros displays specific M4 binding in vitro and additionally induces the recruitment of NFKB1 (NF-Kappa B p50) to M4 WT baits.

**Fig. 8:**
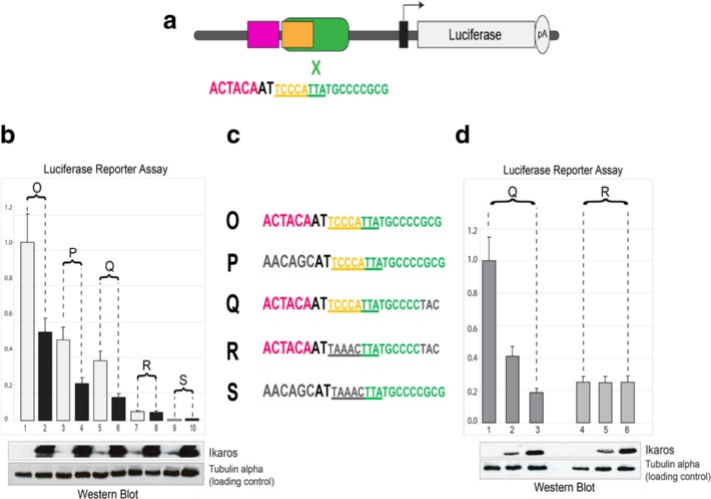
A luciferase M4-reporter assay system to study the functional role of Ikaros for M4-harboring genes. **a** Scheme illustrating the modular M4-reporter gene. The M4 motif is composed of a THAP11 binding site (ACTAYR, *purple*) and an Ikaros binding site (TCCCRR), *yellow*) which overlaps with the binding site of the transcriptional activator STAF/ZNF143 (factor-X in *green*). **b** M4-reporter gene activity (indicated as relative luciferase units) of M4-WT compared to mutated M4-variant promoters in HEK 239 and HEK293-Ikaros cells, which are stably expressing heterologous Ikaros. Transcriptional activity of corresponding M4-promoters in the absence (*black bars*) or presence (*white bars*) of Ikaros. Immunoblot analysis measuring Ikaros protein expression levels and input loading control (tubulin alpha) **c** Nucleotide sequence presentation of the M4-modules and its variants used for M4-reporter gene assays. **d** Transient luciferase reporter assay employing M4-reporter Q and R (details: panel **c**). Ikaros is repressing M4-reporter Q in a dose-dependent manner

To assay the transcriptional activity of an M4-bearing promoter, a well-characterized luciferase reporter (comprising the Xenopus tRNA^Sec^ promoter) was selected, which accommodates a functionally validated binding site for the ubiquitiously expressed transcriptional activator STAF (ZNF143) [24]. The latter fits to the STAF consensus binding sequence (SBS) YWCCCRNMATSCMYYRCR (Y, W, R, N, M, and S stand for T/C, A/T, A/G, any nucleotide, A/C, and G/C, respectively). This sequence was fused at its 5′-position with the sequence of the M4 motif, which is frequently overlapping with SBS in mammalian promoters [24]. Hence, the resulting synthetic promoter sequence (ACTACAATTCCCATTATGCCCCGCG) comprises the following regulatory modules: ACTACA (THAP11-binding site), TCCCATTA (Ikaros-binding site) and TCCCATTATGCCCCGCG (SBS, STAF-binding site; Fig. 8a: depicted as X and highlighted in green). Additionally, each reporter construct contains two copies of the regulatory module that encompasses either the M4 WT motif (Fig. 8c, sequence O and Q) or mutated M4 variants (sequences P, R and S).

As seen in Fig. 8b, Ikaros expression (black bars) correlates with a twofold reduction in luciferase activity (reporter constructs O, P and Q). Importantly, the repressive activity is absolutely dependent on the presence of an intact Ikaros binding site (compare reporter constructs R and S with O, P and Q).

To confirm this observation, the two M4-reporter constructs that harbor mutated (GCG to TAC mutation at the 3′ end) STAF/ZNF143 binding sites, namely reporter Q (intact Ikaros binding site) and sequence R (mutated Ikaros binding site) were selected in order to alleviate the profound transcription stimulatory activity of STAF/ZNF143 (Fig. 8b and c). Then a transient reporter assay was performed expressing increasing amounts of Ikaros (Fig. 8d).

These data (Fig. 8d) clearly demonstrate that Ikaros is repressing reporter gene activity in a strictly dose-dependent manner because Ikaros protein expression level was inversely correlated to transcriptional activity of the M4-reporter Q (Fig. 8d, assays 1 to 3). Conversely, upon Ikaros binding site mutation (M4-reporter R) the repressive effect of Ikaros was completely abrogated (Fig. 8d, assays 4 to 6).

Mutation of the THAP11 binding site diminished reporter gene activity by at least two-fold, both in the presence and absence of Ikaros protein (compare reporter constructs O and P in Fig. 8b).

In summary, in the context of our transient-reporter assays, Ikaros acts as a transcriptional repressor, whereas THAP11 does function as an activator.

### Ikaros knockdown results in de-regulated expression of M4-harboring genes

**Fig. 9:**
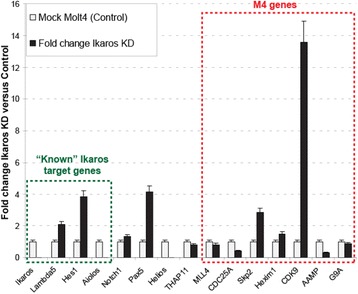
Ikaros loss-of-function (knockdown) leads to de-regulated expression of M4 bearing genes*.* The mRNA level (normalized to GAPDH mRNA amount) fold change of known Ikaros target genes (*green box*) and of selected M4 bearing (*red box*) genes is shown

So far, it was demonstrated that Ikaros is able to regulate the transcriptional output of M4-bearing genes in the context of a transient reporter assay. However, it is not known if Ikaros might play a critical role in gene regulation of endogenous M4-harboring genes. To determine this, the RNA from Molt4 Ikaros knockdown cells (Molt-IK-KD; described above) and Molt4 control cells (Molt4 mock) were used for quantitative RT-PCR experiments in order to monitor the changes in gene expression of selected M4 bearing genes. As seen in Fig. 9, the knockdown of Ikaros did not affect THAP11 mRNA levels significantly. In contrast, it substantially increased the mRNA amount of some well established Ikaros target genes (lacking M4 sequences), like the B cell-specific lambda5 [16] or T cell-specific Hes1-gene, for which Ikaros is known to function as a transcriptional repressor [25, 26]. In parallel, the Ikaros knockdown dramatically reduces the expression level of the Ikaros family gene Aiolos, for which Ikaros serves as an important transcriptional activator [27]. Apart from affecting known Ikaros target genes, depletion of Ikaros caused both up-regulation (Skp2, CDK9 and Hexim1) and down-regulation (CDC25A, AAMP) of selected M4-harboring genes (Fig. 9). In addition, the M4-genes G9a and MLL4 did not virtually change expression. Similar results were obtained from a comparative microarray analysis of Molt-IK-KD versus Molt4-mock cells (data not shown). In conclusion we propose that Ikaros can modulate the activity of M4-harboring genes in living cells.

### THAP11 knockdown leads to weak but significant changes in gene expression of selected M4 motif harboring genes

**Fig. 10:**
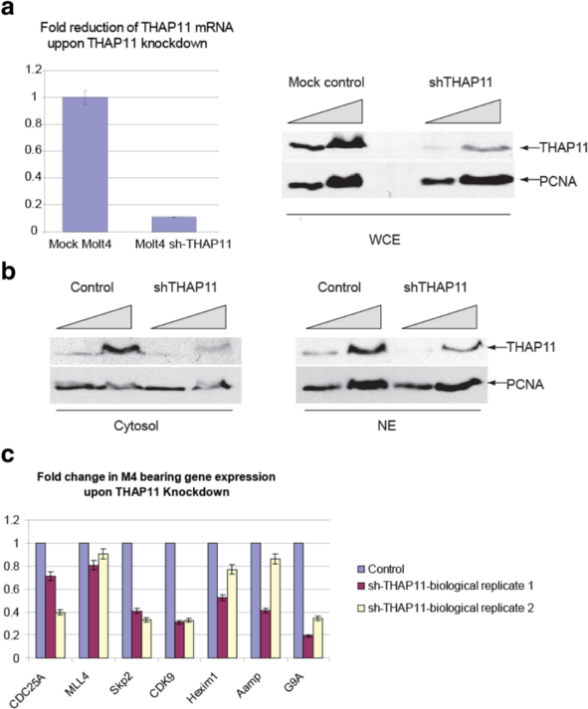
THAP11 knockdown leads to weak but significant changes in selected M4 bearing gene expression. **a** left panel shows fold reduction of THAP11 mRNA upon shRNA-THAP11 knockdown, right panel shows Western blot analysis of THAP11 protein from whole cell extract (WCE) of Molt4 THAP11 knockdown (shTHAP11) or "Mock" Molt4 cells (control); the amount of DNA polymerase delta cofactor PCNA was visualized by its specific antibody and served as SDS-PAGE “loading control”. **b** Cytosol and nuclear extract of Molt4 THAP11 knockdown (shTHAP11) or "Mock" Molt4 cells was subjected to immunoblot analysis to investigate the reduction of THAP11 protein level **c** Fold change of selected M4-gene expression levels upon THAP11 knockdown. Violet bar: M4-gene mRNA levels of control cells (mock Molt4) is normalized to one. *Purple bar*: fold change of M4-gene mRNA levels of Molt4 THAP11 knockdown cells extracted right after establishing the shTHAP11 clone (biological replicate 1). *Yellow bar*: fold change of M4-gene mRNA levels measured from biological replicate 2 (mRNA preparation 2 weeks after biological replicate 1) of Molt4 THAP11 knockdown cells

Finally, for deciphering the biological and functional relevance of the THAP11-M4 motif interaction in living cells, a stable (shRNA) THAP11 knockdown cell was generated (Molt4-sh-THAP11). However, it seems that THAP11 might be essential for cell viability, because selection of clones that exhibited both a complete ablation of THAP11 mRNA levels and a substantial decrease in THAP11 expression was not possible (Fig. 10a). It was noted that THAP11 localizes to both the cytoplasm and nucleus (Fig. 10b and data not shown). Surprisingly, the knockdown mainly affected its cytosolic fraction while keeping a reduced, but still substantial amount of THAP11 in the nucleus (Fig. 10b). As for this reason, strong effects of the THAP11 knockdown on M4 target gene expression were not expected. To provide reproducible data it was decided to conduct biological replicate experiments in which the extraction of Molt4-sh-THAP11 total mRNA was carried out at two different time points (with a time gap of 2 weeks) after selecting the clone. These samples are termed biological replicate 1 and biological replicate 2, respectively.

As seen in Fig. 10c, the biological replicate 1 (red bars) of Molt4-sh-THAP11 transcript exhibits a ten-fold reduction of THAP11 mRNA that correlates with the down-regulation of most selected M4 bearing genes. This is in accordance with previous observations made in transient reporter assays (Fig. 9) and suggests that THAP11 works as a transcriptional activator in controlling M4 motif-bearing genes. Two weeks after the first mRNA extraction, biological replicate 2 (yellow bar) of Molt4-sh-THAP11 mRNA preparation was performed. The quantitative PCR (qPCR) analysis of the second mRNA extraction revealed reproducible effects of THAP11 protein reduction on the transcript levels of CDC25A, Skp2, CDK9, Mll4, and G9a, whereas the influence of THAP11 on gene expression levels of Hexim1 and AAMP was quite variable.

## Discussion and conclusions

The motif M4 ACTAYRNNNCCCR (Y being C or T, R being A or G, and N as any nucleotide) was discovered by Xie and colleagues in 2005 [7] as a putative cis-regulatory element. This motif was described to be highly conserved in a window from less than 2500 bps upstream and 500 bps downstream of transcription start sites (TSSs) of many annotated genes of human, mouse, rat, and dog with a peak centered around − 89 bp from TSSs. Hence, the hyperconserved M4 motif can be considered as a core-promoter associated cis-element that can be found on 520 human genes, implying a potential important role in regulating multiple cellular processes. Recently, by using ChIP-Seq assays combined with bioinformatics, two independent groups [9, 11] have demonstrated M4 motifs as binding site for RBP-J and ICN1 (activated Notch1), as well as STAF/ZNF143, and THAP11 (also known as Ronin in mouse), mostly in context of non-lymphoid cells like HeLa, HEK293, K562 and murine ES cells. However, according to the initial publication by Xie and colleagues, genes bearing the M4 motif are preferentially expressed in human cells of hematopoietic origin (e.g. B lymphoblasts, peripheral blood CD19 B cells as well as CD4 and CD8 T cells), which prompted us to revisit and comprehensively characterize the M4 TF binding landscape in lymphoid cell lines.

**Fig. 11:**
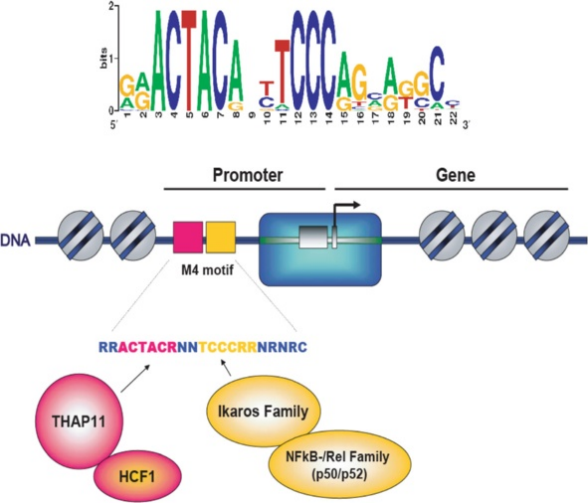
Working model for the assembly of M4 binding factors on promoters of M4 genes. The M4 motif is composed of two highly conserved elements: an “upstream” ACTACR module (*purple*) and a “downstream” module TCCCRR (*yellow*), which constitute bipartite binding sites for THAP11 and Ikaros, respectively. Interaction between THAP11 and HCF-1 is required for recruiting HCF-1 to the M4 motif, whereas an interaction of Ikaros with NFKB1 (NF-kappa B p50) or NFKB2 (NF-kappa B p52) is essential for tethering these Rel TF family proteins to the M4 motif

In order to pursue this we employed our SILAC-based DNA protein interaction screening method, which we showed to be technically capable of uncovering direct and indirect (“piggy-back”) TF cis-element interactions [4]. In this respect, our motivation was twofold. First of all, we wanted to make use of the partially studied M4 model with the aim of demonstrating that our quantitative proteomic methodology [4] is working as a surrogate for a bona fide reverse ChIP technology. Second, we intended to screen for M4-binders in an unbiased manner exclusively utilizing the M4 sequence logo [28] information derived from Xie and colleagues [7], thereby avoiding issues with the overlap of M4 with SBS (STAF/ZNF143 Binding Sequence [9]). Building on that, in this proteomic study, we successfully identified the nuclear factors THAP11, HCF-1, Ikaros, Aiolos, Helios, NFKB1 (NF-kappa B p50) and NFKB2 (NF-kappa B p52) as interacting partners of the M4 motif in the context of a human lymphocyte nuclear extract (Figs. 2 and 3). Limited by the availability of reagents we conducted follow-up experiments for THAP11, HCF-1, Ikaros and NFKB1. Importantly, we demonstrated that at least in lymphocyte cell lines, M4 is functioning as a bipartite composite element, in which the “upstream” ACTAYR part constitutes a binding site for the TF THAP11, while the “downstream” CCCR module provides the direct DNA contact for the lymphocyte specific TF Ikaros. Most probable and similar to Ikaros, Helios and Aiolos might also play a modulatory role for certain M4 genes, but it is very difficult to investigate their function because Ikaros itself serves as a master regulator of Aiolos and Helios gene transcription (Fig. 9, [27]). Nevertheless, it would be interesting to investigate this further because Ikaros and Helios can have opposite functions at the same target gene [29]. In our model (Fig. 11) and building on our data discussed below the epigenetic factor HCF-1 is recruited to M4 motif DNA by virtue of its interaction with the THAP11 C-terminal domain (Additional file 1: Figure S3), whereas the Rel/NF-kappa B-factors (NFKB1/NF-kappa B p50 or NFKB2/NF-kappa p52) are strongly believed to be tethered to the M4 element in an Ikaros-dependent manner (Fig. 6). These findings indeed verify the power of the SILAC-based DNA protein interaction screening methodas a comprehensive approach, which can capture direct and indirect DNA-TF assemblies. In contrast, the limitation of EMSA is seen in Fig. 1, for which EMSA was carried out with Raji nuclear extracts. The latter showed at least two strongly retarded (shifted) bands that are virtually absent in HeLa nuclear extracts, albeit THAP11 and HCF-1 are equally well expressed in HeLa cells (data not shown). This is surprising because bacterially expressed THAP11 [9] is able to retard radioactive-labelled DNA sequences bearing the M4 motif in conjuction with the SBS element. This phenomenon could be explained by the lack of lymphocyte specific factors in Hela nuclear extracts that together with THAP11 and HCF-1 might be responsible for higher order complex formation.

Ikaros and NF-kappa B proteins are direct DNA binding factors whose canonical binding or half sites [17] are similar to the CCCR module of M4 motifs. However in nuclear extracts prepared from cells that do not express Ikaros (HEK293, HeLa) the binding of NFKB1 (NF-kappa B p50) to M4 could not be detected. Interestingly, the binding of NFKB1 to the “downstream” module of M4 was clearly restored upon heterologous expression of Ikaros in HEK293 cells (Fig. 6c), suggesting an essential role of Ikaros protein in tethering NF-kappa B proteins to M4. This was further corroborated in experiments that utilized an Ikaros knockdown Molt4 cell line, which does not express any Ikaros as assessed by western blotting (Fig. 6a). Employing nuclear extracts from these KD cells revealed that recruitment of NFKB1 (NF-kappa B p50) to M4 DNA was completely abrogated, while binding of THAP11 and HCF-1 was essentially not affected (Fig. 6a). Beyond this, Ikaros KD cells do also loose expression of Helios and Aiolos at least at the mRNA level (Fig. 9). Therefore, it will be interesting to test in future experiments if Helios and Aiolos are also capable to promote NFKB1 binding to M4. The observations discussed above were further recapitulated by ChIP assays performed in Molt4 Ikaros KD cells. More precisely, the presence of NFKB1 (NF-kappa B p50) at M4 motif harboring promoters was severely compromised (Fig. 7c), whereas recruitment of THAP11 and HCF-1 was still preserved (Fig. 7a and b).

It could therefore be inferred that NFKB1 (NF-kappaB p50) is recruited to M4-genes in an Ikaros dependent manner. Mechanistically, this is probably taking place by the formation of Ikaros-NFKB1 protein complexes that we detected by coimmunoprecipitation of Ikaros employing an anti-NFKB1 antibody and copurification of NFKB1 (NF-kappa B p50) with StrepII-tagged Ikaros, respectively (Fig. 6c and d). Since we did not carry out in vitro binding studies with recombinant Ikaros and NFKB1 proteins it is formally still possible that their interaction is either indirect or heavily dependent on the presence of M4 containing DNA/chromatin. Furthermore, we cannot rule out the possibility that Ikaros KD has an additional influence on NF-kappa B signaling, eventually resulting in a decreased ability of NFKB1 to bind to DNA/chromatin (Fig. 7d). Based on the results obtained from reporter assays and Ikaros loss-of-function (Ikaros KD) experiments we are confident that the association of Ikaros with hyperconserved M4 elements is functionally relevant (Figs. 8 and 9). However, an elucidation of the detailed mechanistic action of Ikaros (and NFKB1) in governing the transcriptional output of M4 bearing genes might be gene-specific [30] and was outside the scope of this manuscript. As described for the negative cross-talk between STAF/ZNF143 and RBP-J-ICN1 heteroconjugates [9], Ikaros-NFKB1 complexes could similarly compete with M4 binding of STAF/ZNF143 and/or RBP-J, especially in light of the fact that at the genome-wide level Ikaros and RBP-J are generally sharing the same short binding consensus [31]. Together this could also mean that the transcriptional output of M4 genes can be modulated by a complex interplay of relative expression levels of Ikaros, Helios, Aiolos and RBP-J that is further orchestrated by the status of Notch1 and NF-kappa B pathway signaling inputs. At this instance, it has been reported that NFKB1 (NF-kappa B p50), which in contrast to RELA/NFKB3 (NF-kappa B p65) is lacking a classical transactivation domain, can be found in N-CoR corepressor complexes. Upon interleukin-1 beta stimulation the promoter occupancy of N-CoR corepressor complex at NFKB1 bound promoters is abolished, which is followed by recruitment of Tip60 coactivator complexes and a subsequent change in the gene expression program [32, 33]. Interestingly, our Ikaros interactome from Molt4 cells (unpublished results) contains various corepressors (RCOR1/CoREST, RCOR3, NuRD) along with NFKB1 and NFKB2, while THAP11 and HCF-1 are absent.

Collectively, we would like to propose the current working model for M4 function in lympocytes that is depicted in Fig. 11. In accordance to previous findings [10, 11] THAP11, whose binding is required for the recruitment of HCF-1 binds directly to the “upstream” ACTACR module of the M4 motif. Conversely, Ikaros interacts with the “downstream” module (YCCCR) and is either directly or indirectly responsible for the association of the Rel family TFs NFKB1 (p50) and NFKB2 (p52). As discussed above other TFs like STAF/ZNF143 and RBP-J (recruiting activated Notch1-ICN1) [9] whose binding sites do also comprise the “downstream” module YCCCR would also bind to M4 in non-lymphoid cells.

## Methods

### M4 motif bearing DNA ligand preparation

100 nM sense 5 prime biotinylated oligonucleotide (synthesized by Eurofins MWG Operon) bearing either wild-type or mutant M4 motif were combined with 110 nM anti-sense oligonucleotide in 200 ul 20 mM Tris-HCl, pH 8.0, 200 mM NaCl, 0.5 mM EDTA, 0.03 % NP40. The reaction mixture was heated at 95 °C for 5 min and then cooled down in a stepwise manner to 65 °C over 15 min, and kept at 65 °C for 5 min before the reaction is allowd to be cooled to RT. At this point, the annealed double stranded oligo-nucleotide can either be stored at − 20 °C for long-term use or used in the following step.

### Immobilization of biotinylated oligonucleotide to the beads

100 μl suspension of Dyanabeads® Myone Streptavidin bead (Dynal Biotech ASA, Oslo, Norway) was combined with 13 μl annealed M4 motif bearing DNA ligand in 400 μl 20 mM Tris-HCl, pH 8.0, 200 mM NaCl, 0.5 mM EDTA, 0.03 % NP40 for 3 h at room temperature and then rotated at 4 °C overnight. Afterwards, the beads were washed by three volumes of 800 μl 20 mM Tris-HCl, pH 8.0, 200 mM NaCl, 0.5 mM EDTA, 0.03 % NP40 buffer, and re-suspended in 100 μl of the same buffer. At this point, the concentration of DNA ligand on bead suspension was approximately 2.73 nM and could be stored at 4 °C for later use.

### One step-DNA affinity chromatography

The human T lymphoblastic leukemia Molt 4 or Raji burkitt’s lymphoma cells were metabolically labeled in a RPMI medium supplemented with either light or heavy isotopes of two essential amino acids (^1^H_4_ lysine and ^12^C_6_ arginine) or (^2^H_4_ lysine and ^13^C_6_ arginine) or just (^2^H_4_ lysine) as described before [34]. After five doubling cycles, the cells were expected to be more than 98 % isotopically-encoded. Two separate populations of isotope-encoded cells were equally expanded to 5 l of 1 × 10^6^ cell/ml and subjected for crude nuclear extract preparation. The crude nuclear extract was then dialyzed at 4 °C against a buffer (20 mM Tris-HCl, pH 7.3 containing 100 mM KCl, 20 % glycerol and fresh protease/phosphates inhibitors) and frozen in liquid nitrogen for further use.

Three volumes of crude nuclear extract from 1.5x10^8^ cells were then incubated with three vials of magnetic beads immobilized with 40nM M4 motif DNA ligand in the buffer (20 mM Tris-HCl, pH 7.3 containing 100 mM KCl, 10 % glycerol and fresh protease/phosphates inhibitors) for 4 h, rotating at 4 °C. After washing away the unbound materials, protein-DNA complexes were then librated by PstI endonuclease restriction digestion.

### Liquid chromatography mass spectrometry

Proteins eluted from the DNA affinity columns were resolved through 4–12 % Bis-Tris SDS gels (NuPAGE, Invitrogen) and stained with colloidal Coomassie. The gels were sliced into 10 equally sized gel pieces and subjected to tryptic in-gel digestion [35]. Prior to LC-MS analysis, tryptic peptide mixtures were desalted using STAGE tips (Empore high performance extraction disks C18–IVA Anlysentechnik e. K Meerbusch, Germany) as described previously [36] and the pooled elutates from the C18 tips were subsequently analyzed by nanoscale-LC (Agilent 1200 nanoflow system), coupled either to a Hybrid LTQ OrbitrapXL or LTQ-FTUltra mass spectrometer (Thermo Fisher Scientific Inc. Bremen, Germany).

Peptides were eluted from an analytical column by a linear gradient running from 5 to 80 % (v/v) acetonitrile (in 0.5 % acetic acid) with a flow rate of 250 nl/min in 120 min gradient and sprayed directly into the orifice of the mass spectrometer. The 15 cm fused silica emitter with an inner diameter of 75 μm (New Objective, USA) was packed in-house with reverse phase ReproSil-Pur C18–AQ 3 μm resin (Dr. Maisch GmbH, Germany). Data dependent acquisition of MS, MS/MS was performed: Fullscan MS spectra (m/z 350–1600) were acquired in the Orbitrap detector with resolution R = 60,000 at m/z = 400 with a target value of 1,000,000 ions allowing a maximum injection-time of 1200 ms. The five most intense ions were sequentially isolated for SIM (selected ion monitoring), within an isolation width of 2.0 and a target accumulation value of 50,000 (maximum fill-time = 500 ms). 30,000 ions were fragmented in the linear ion trap by CID (collision induced dissociation) at a target value of 10,000 (maximum fill-time = 50 ms). Target ions selected for MS2 were dynamically excluded for 180 s. The general mass spectrometric conditions were: spray voltage, 2.4 kV; no sheath and auxiliary gas flow; capillary temperature, 120 °C; normalized collision energy 35.0, Ion selection thresholds were 500 counts for MS2. An activation q = 0.25 and activation time of 30 ms was applied for MS2 acquisition.

### Data analysis

The raw Orbitrap MS files were processed with MSQuant v2.0b5 and MaxQuant v1.0.12.31 [37, 38] two powerful computational platform for SILAC based quantitative proteomics, using the following settings: Enzyme specificity was set to trypsin, allowing two total missed cleavages, at N-terminal to proline segments, and between aspartic acid and proline. Carbamidomethylation of cysteine were set as a fixed modification, while methionine oxidation, protein N-acetylation, and loss of ammonia from glutamine and asparagine were set at variable modifications. The peak lists generated by MaxQuant were searched with an in-house Mascot 2.2 server against the human international protein index (IPI) database (v.3.64) containing frequently observed contaminants concatenated with a decoy of the reversed sequences. For monoisotopic precursor ions, the maximum allowed mass deviation was set to 5 ppm and for MS/MS peaks to 0.5 Da. The identified proteins and peptides were further processed with MaxQuant (imported as Mascot .dat files) with a minimum required peptide length of six amino acids and a false discovery rate setting of 0.01 at the protein level. For protein identification and quantification, two unique peptides and two quantified peptides were necessary. Before statistical analysis, known contaminants were removed.

### Ectopic expression retroviral transduced cell lines

Human cDNA coding for Ikaros was tagged at N terminal with a StrepII-tandem-tag or HA epitope; the HA epitope was also used to tag cDNA sequence coding for human THAP11 at its C-terminal. These two chimeric cDNA were PCR cloned into pMXs-IP retrovirus plasmid [39] (the primers used for cloning could be found in supplimentary study data) and used for generating the stable cell line Molt4-Strep-flag-Ikaros or Molt4-Ikaros-HA or Molt4-THAP11-HA cell lines as describling previously [39, 40].

### Chromatin immunoprecipitation

50 ml Molt4 cell aliquots of 5×10^5^cells/ml were treated with 1 % formaldehyde (final concentration) at RT for 5 min and then with 10 mM Disuccinimidyl glutarate (final concentration) for another 2 min at RT. The cross-linked cells were then washed 3 times with cold PBS and resuspended in 850 μl cold-L1 lysis buffer (50 mM Tris–HCl, pH 8.0, 2 mM EDTA, 0.1 % NP40, 10 % Glycerol, 2 mM fresh DTT, fresh protease inhibitors, fresh phosphatase inhibitors, 1 mM sodium butyrate) for 10 min on ice. The cells were spun down at 800×g for 5 min to clear out the cytoplasm fraction (supernatant) and the pellet (cross-linked nuclei) were re-suspended in 850 μl cold-L2 lysis buffer (50 mM Tris-HCl, pH 8.0, 2 mM EDTA, 1 % SDS, 2 mM fresh DTT, fresh protease inhibitors, fresh phosphatase inhibitors, sodium butyrate). At this point, the cross-linked nuclei can be snapped down in liquid nitrogen and stored at − 80 °C. Otherwise, the procedure can be continued by sonifying the cross-linked nuclei, shearing the chromatin into pieces of 300–1000 bp length, and used as the input for immune-precipitation:

The sheared chromatin amount corresponding to one aliquot of cross-linked nuclei (2.5 × 10^6^ cells) was then diluted 10 times into 1.5 ml DB buffer (50 mM Tris-HCl, pH 8, 5 mM EDTA, 200 mM NaCl, 0.5 % NP40, 2 mM fresh DTT, fresh protease inhibitors, fresh phosphatase inhibitors, sodium butyrate) and incubated with 2.5.0 μg rabbit Ikaros antibody [41] or rat anti-HA antibody (3 F10 clone-Roche) which target the HA epitope of Ikaros or THAP11 protein or HCF-1 antibody [10], or NFkB p50 antibody (H-119-Santa Cruz Biotech) or NFkB p52 antibody (C-5-Santa Cruz Biotech) or the in-house raised rat monoclonal antibody that targets an epitope at the C-terminus of THAP11 protein for 5 h. The control assay was carried out at the same time using 2.5 μg rabbit pre-immune-serum and equal amounts of input material. The DNA-protein complexes were precipitated by adding 30-40 μl salmon sperm, saturated with protein G. The incubation time was extended for 45 min and the beads were then washed three times with a NaCl washing buffer (20 mM Tris-HCl, pH 8, 2 mM EDTA, 500 mM NaCl, 1 % NP40, 1 % SDS) and once with a LiCl washing buffer (20 mM Tris-HCl, pH 8, 2 mM EDTA, 500 mM LiCl, 1 % NP40, 1 % SDS). The G protein bound materials were released by incubating with 2 volumes of 150 μl 20 mM Tris-HCl buffer pH8 containing 2 % SDS and 0.5 mM EDTA for 5 min at 65 °C. The released IP DNA was purified by using a Qiagen PCR cleaning kit and stored in 250 μl 20 mM Tris-HCl buffer pH8 0.5 mM EDTA at − 20 °C for later realtime qPCR analysis.

### Realtime qPCR analysis

2 μl of IP DNA or input DNA was templated for sybr-green-based-realtime-qPCR reactions using primer pairs as indicated in Table 1. Enrichment ratios of individual M4 motif bearing promoters were calculated by normalizing the qPCR signal from IP DNA templates against the signal from corresponding input genomics DNA.

## Additional file

Additional file 1: Does contain additional **Figure S1**, additional **Figure S2** and **Figure S3**. (DOC 802 kb)

## Abbreviations

ChIP: Chromatin immunoprecipitation
EMSA: Electrophoretic mobility shift assay
GST: Glutathione S-transferase
HA: Hemagglutinin
HCF-1: Host cell factor-1
HEK293: Human embryonic kidney (cells)
IP: Immunoprecipitation
KD: Knockdown
LC-MS: Liquid chromatography mass spectrometry
MT: Mutant
qRT-PCR: Quantitative real time PCR
SBS: STAF/ZNF143 Binding Site
shRNA: Short hairpin RNA
SILAC: Stable isotope labeling with amino acids in cell culture
STAF: Selenocysteine tRNA gene transcription activating factor
TF: Transcription factor
THAP11: Thanatos associated protein 11
TSSs: Transcription start sites
WT: Wild-type
ZNF143: Zinc finger protein 143 (alternative name: STAF)
