# Supplementary material for: Biochemical and cellular characterization of transcription factors binding to the hyperconserved core promoter-associated M4 motif
Source: BMC Genomics. 2016 Aug 30;17(1):693. doi: 10.1186/s12864-016-3033-3 (PMC5006282; doi:10.1186/s12864-016-3033-3)
Supplement: Additional file 1: — Does contain additional Figure S1, additional Figure S2 and Figure S3. (DOC 802 kb) [file 12864_2016_3033_MOESM1_ESM.doc]

# Biochemical and cellular characterization of transcription factors binding to the hyperconserved core promoter-associated M4 motif

# Ngo Tat Trung^1,2,3^, Elisabeth Kremmer^4,5^ and Gerhard Mittler^1,6*^

# 1) Department of Cellular and Molecular Immunology, Proteomics Core Facility, Max-Planck-Institute of Immunobiology, D-79108 Freiburg, Germany

# 2) [Albert-Ludwigs-University Freiburg](http://www.uni-freiburg.de/universitaet-en), Faculty of Biology, Schänzlestrasse 1, D-79104 Freiburg, Germany

# 3) Current address: Tran Hung Dao University Hospital (Benh Vien TWQD 108), No 1, Tran Hung Dao Str, Hai Ba Trung Dist, Hanoi, Vietnam

4) Institute of Molecular Immunology, Helmholtz Center Munich, German Research Center for Environmental Health (GmbH), D-81377 München, Germany

5) Current address: Ludwig-Maximilians-University Munich, Biocenter, Humanbiologie und Bioimaging, D-82152 Martinsried, Germany

6) BIOSS, Center for Biological Signalling Studies, University of Freiburg, Schänzlestr. 18, D-79104 Freiburg, Germany

# *) Corresponding author

# Email addresses:

# Ngo Tat Trung: [**Trungnt@benhvien108.vn**](mailto:Trungnt@benhvien108.vn)

# Gerhard Mittler: [**mittler@ie-freiburg.mpg.de**](mailto:mittler@ie-freiburg.mpg.de)

**Additional figure 1***: Ectopically expressed Ikaros-HA and THAP11-HA (bearing a C-terminal HA-tag) interact with mono M4 motif in vitro: DNA affinity chromatography experiments (“DNA pulldown”) with immobilized mono-M4 WT and mono M4 MT DNA as a ligand were performed as follows. Nuclear extracts from Molt4-Ikaros-HA or Molt4-THAP11-HA cells served as inputs. Corresponding column fractions were subjected to Western blot analysis. Anti HA antibody was used to detect heterologous expression of Ikaros-HA or THAP11-HA fusion proteins. The two unspecific binders IFI-16 and TPB were visualized by specific antibodies and served as SDS-PAGE “loading controls”. Abbreviations: FT‑WT M4 (flow-through M4 wild‑type), FT-M4-MT (flow-through M4‑mutant), E M4-WT (eluate M4 wild‑type), E M4 –MT (eluate M4‑mutant).*

**Additional figure 2**: **Ectopically expressed StrepII-Flag-Ikaros specifically and selectively interacts with M4 motif containing gene promoters in vivo***. Left panel: human cDNA coding for Ikaros was cloned and N‑terminally tagged with a StrepII-tandem-tag/Flag-tag double epitope and used to generate the stable cell line Molt4-Strep-flag-Ikaros. Right panel: X-ChIP experiments were performed essentially as described in figure 24 except that Streptactin columns (recognizing the StrepII-tandem-tag) substitute for the antibody protein A/G sepahrose beads. Standard Molt 4 cells not expressing StrepII‑tagged Ikaros served as a specificity control (Molt control, open bars). Enrichment of TF-bound DNA amount was normalized to corresponding amount in input.*

**Additional figure 3:** *GST pull-down assay confirms THAP11-HCF-1 interaction. GST (control), the GST‑THAP11 C‑terminus and GST‑THAP11 were expressed in and purified from E. coli (lower panel). Affinity pull-down assays employed recombinant GST, GST-THAP11-C-terminus and GST-THAP11 immobilized as ligands on glutathione-sepharose beads in conjunction with Molt4 nuclear extracts that served as input. ,After washing away unbound material, the bound protein complexes were eluted (E GST, E GST‑THAP11-C-term, E GST‑THAP11), seperated by SDS PAGE and tested by western bloting conducted with a HCF‑1-specific antibody (upper panel). Note that HCF‑1 is expressed as a precursor protein in mammalian cells that is proteolytically processed to form a dimer comprising the N‑‑and C‑terminal fragments.*

# Additional materials and methods

## Primer sequences used in transcript quantification by real time qPCR

| Gene names | Acession Number | Primer sequences (Forward/Reverse from 5’to 3’) |
| --- | --- | --- |
| CDC45 | NM_001254 | cctgttctcctcgtgtaaaagc/gtgttgcataggttgtcatcg |
| MAX | NM_145116 | ccagcaagatattgacgacct/agctcgggtgctcacctt |
| PRIM1 | NM_000946 | gcatcattgacagagcattga/tcacagacccaacaatgaaca |
| MML4 | NM_014727 | ccagacctgctgcttgagt/ctcagagctcgaagcctcac |
| AAMP | NM_001087 | aagaggaggaagagggcaac/tcacacaaaacacagatgctga |
| CDC25A | NM_201567 | ctccgagtcaacagattcagg/ttcaaggttttctttactgtccaa |
| Skp2 | NM_005983 | ctgtctcaaggggtgattgc/ttcgataggtccatgtgctg |
| G9A( EHMT2 L) | NM_025256 | gggaccttcatctgcgagta/cctctctcacatcagcctca |
| CDK9 | NM_00126 | ttcggggaggtgttcaag /atctcccgcaaggctgtaat |
| Igll1 | [NM_020070](http://www.ncbi.nlm.nih.gov/entrez/viewer.fcgi?db=nuccore&id=23110977) | gaatgacttttatccgggaatct/ggtgatgggggtaccatct |
| E2N | NM_003348 | ctcgggttctgacaagatgg/ctcatctggttcggctttg |
| Notch1 | NM_017617 | cgcacaaggtgtcttccag/aggatcagtggcgtcgtg |
| TBP | NM_003194 | gctggcccatagtgatcttt/cttcacacgccaagaaacagt |
| Il2 | NM_000586 | aagttttacatgcccaagaagg/aagtgaaagtttttgctttgagc |
| MLL2 | NM_003482.2 | ggcaaaggaagtgaggtgtc/catcacgccattcaggttc |
| Hexim1 | NM_006460 | gcagcgtcatcggtagtttt/agggtaagtccagtgcgtga |
| IKZF1(Ikaros) | [NM_006060.3](http://qpcr.probefinder.com/showsequence.jsp;jsessionid=E6EC0019E66551526918F1B317CFAC34?seqNo=1509718305) | ccttccgggcacactgta/tctctctgatcctatcttgcaca |
| THAP11 | [NM_020457.2](http://qpcr.probefinder.com/showsequence.jsp;jsessionid=E6EC0019E66551526918F1B317CFAC34?seqNo=1245363669) | gcctctgcctcctaggactt/ggtcatccagatccccttg |
| IKZF3(Aiolos) | NM_012481 | aatgcggaactgaaaagcac/tcaaaaccgctgcactttct |
| IKZF2(Helios) | NM_016260 | tctccagaatgtcagcatgg/tccttacaatcttccataggaggta |
| HES1 | NM_005524 | agtgaagcacctccggaac/cgttcatgcactcgctga |
| Pax5 | NM_016734 | acgctgacagggatggtg /cctccaggagtcgttgtacg |

## Primer used in quantification of immuno-precipitation chromatin.

| Gene names | Position (relative to M4 sequence) | Primer sequences (forward/reverse from 5’ to 3’) |
| --- | --- | --- |
| Skp2 | M4 flanking region | gaagcgggacggaaacta/tctacagcccgctctgct |
| Cdc25a | M4 flanking region | gcgatgggaactacgcttc/gggagcagaggtcaatgaaa |
| G9a | M4 flanking region | ctgcccacggaaattgtaag/aaaggccttctgggaactgt |
| Skp2 | 5’ control region | tcaagaacgagctaaaggttca/gcttggtccttagcttgaaaag |
| Cdc25a | 5’ control region | ccagcaaccttgacctttagttt/tggtgggcacctagagga |
| G9a | 5’ control region | gaccacctcaacctgcactc/ttgaggtgccgcctaatg |
| Skp2 | 3’ control region | gtgctaggccgtgttcca/gctccctgagagcagaaatg |
| Cdc25a | 3’ control region | ccctgtgcacattcctgat/tgaaagttgggtaaaaagacctg |
| G9a | 3’ control region | gagtttggctatgaggctactga/gctggagggggttcagac |
| Skp2 | exon control region | tgtctcttctttagaacagggaaaa/atgaaggcaaagggaaaacc |
| Cdc25a | exon control region | tcgtctgaagaagctctgagg/agagggtaaagggggatgg |
| G9a | exon control region | ccaccaccctttcacacatt/ccagggaggaaccagctc |

## Primers used for EcoRI/XhoI cloning Ikaros into pMX-IP system

## Forward: ttttttggatccctgaggaccaccatggacatggatgctgatgagggtc

Reverse:

Gaattcttaagcgtaatctggaacatcgtatgggtactcgagagcgtaatctggaacatcgtatgggtagctcatgtggaagcggtg

## Primers used for EcoRI/XhoI cloning THAP11 into pMX-IP system

Forward: tattggaattc accatgg atgcctggctttacgtgct

Reverse: tactcgagttaagcgtaatctggaacatcgtatgggtacattccgtgcttcttgcg

## Oligo-nucleotides used in EMSA assay:

BTRC WT up: 5’-tcctgggggaagttccagaactacaaatcccgtgagccagtgggctttcgc – 3’

BTRC WT down: 5’-cgaaagcccactggctcacgggatttgtagttctggaacttcccccagga – 3’

Underlined sequences are the M4 motif. All of the BTRC oligo-nucleotides were synthesized by DNA Technology A/S Denmark and delivered in lyophilized status. We reconstitute them in 1x annealing buffer to get the concentration of 110 nM.

## Oligo used in proteomic screening to identify M4 interacting factors

Tandem M4 WT

Caaaactgcaggcaactagaactacatttcccggcgtgatttgctgcggaacgaactacatctcccggcaggctgcg

Tandem M4 MT

Caaaactgcaggcaactagacatcaatttctgggcgtgatttgctgcggaacgacatcaatctctgggcaggctgcg

(The red letters indicate M4 motif)

## DNA sequences used as promoters in reporter assay

Tandem M4 wild type (M4A):

ctcgagctctgacgcggaaactctgtgtgactcaggctgacccccattgtcactgtactacaattcccattatgccccgcgaagaccaacagcagaccagcgcagtgatgtatcccacatccactaacaaacagcaaccacacgcccctcctccctcttcccgtttttcattgaaaataaaccgaagcgtattgtactacaattcccattatgccccgcgcgcgcgtgtatgctatgcgatcttgtttgatttccctcgatttcacgttagactagtcggggtatgtaagcggcgatacgtttaactagaaaaggaatagtcagatttttgataccagaaacaattttttgtcaccccaatatataatataatggtgggagggggtataaaaggaaatgggagtactgaccatgg

Tandem ΔTHAP11 M4 (M4B):

Ctcgagctctgacgcggaaactctgtgtgactcaggctgacccccattgtcactgtaacagcattcccattatgccccgcgaagaccaacagcagaccagcgcagtgatgtatcccacatccactaacaaacagcaaccacacgcccctcctccctcttcccgtttttcattgaaaataaaccgaagcgtattgtaacagcattcccattatgccccgcgcgcgcgtgtatgctatgcgatcttgtttgatttccctcgatttcacgttagactagtcggggtatgtaagcggcgatacgtttaactagaaaaggaatagtcagatttttgataccagaaacaattttttgtcaccccaatatataatataatggtgggagggggtataaaaggaaatgggagtactgaccatgg

Tandem Modified M4 (M4C):

Ctcgagctctgacgcggaaactctgtgtgactcaggctgacccccattgtcactgtactacaattcccattatgcccctacaagaccaacagcagaccagcgcagtgatgtatcccacatccactaacaaacagcaaccacacgcccctcctccctcttcccgtttttcattgaaaataaaccgaagcgtattgtactacaattcccattatgcccccgtcgcgcgtgtatgctatgcgatcttgtttgatttccctcgatttcacgttagactagtcggggtatgtaagcggcgatacgtttaactagaaaaggaatagtcagatttttgataccagaaacaattttttgtcaccccaatatataatataatggtgggagggggtataaaaggaaatgggagtactgaccatgg

Tandem ΔIkaros Modified M4 (M4D):

ctcgagctctgacgcggaaactctgtgtgactcaggctgacccccattgtcactgtactacaattaaacttatgcccctacaagaccaacagcagaccagcgcagtgatgtatcccacatccactaacaaacagcaaccacacgcccctcctccctcttcccgtttttcattgaaaataaaccgaagcgtattgtactacaattaaacttatgcccccgtcgcgcgtgtatgctatgcgatcttgtttgatttccctcgatttcacgttagactagtcggggtatgtaagcggcgatacgtttaactagaaaaggaatagtcagatttttgataccagaaacaattttttgtcaccccaatatataatataatggtgggagggggtataaaaggaaatgggagtactgaccatgg

Tandem ΔTHAP11- ΔIkaros M4 (M4E):

Ctcgagctctgacgcggaaactctgtgtgactcaggctgacccccattgtcactgtaacagcattaaacttatgccccgcgaagaccaacagcagaccagcgcagtgatgtatcccacatccactaacaaacagcaaccacacgcccctcctccctcttcccgtttttcattgaaaataaaccgaagcgtattgtaacagcattaaacttatgccccgcgcgcgcgtgtatgctatgcgatcttgtttgatttccctcgatttcacgttagactagtcggggtatgtaagcggcgatacgtttaactagaaaaggaatagtcagatttttgataccagaaacaattttttgtcaccccaatatataatataatggtgggagggggtataaaaggaaatgggagtactgaccatgg

The synthetic tandem M4 wild type was derived from Xenopus Selenocysteine tRNA[Ser]Sec gene promoter, underlined letters are M4 sequences

The Tandem ΔTHAP11 M4 (M4B) was designed by exchanging actaca for aacagc thereby abolishing the binding of M4 motif to THAP11but maintaining Ikaros binding site intact.

To assay the transcriptional activity of M4 and M4 binding factors only but the sequence actacaattcccattatgccccgcg is naturally a composite of M4 motif and the overlapped Staf binding site. Therefore, the sequence gcg at very downstream of M4 motif was modified into tac that minimize the influence of transcription factor Staf on regulating tamdem M4 promoter.

To assay the activity of Ikaros only on driving the tandem M4 motif habouring promoter, we mutate the Ikaros binding site of Tandem Modified M4 (M4C) sequence, but keeping THAP11 binding site intact. As seen in the sequence Tandem ΔIkaros Modified M4 (M4D), ttcccatt of M4 motif is exchanged for ttaaactt

As the full control for the transcriptional activity of M4 motif, the sequence Tandem ΔTHAP11- ΔIkaros M4 (M4E) was designed by mutating the binding sites of both THAP11 and Ikaros.

### Immuno-precipitation (IP assay):

The mammal cell line of interest is grown up to 5x10^5^cells/ml and was washed two times by cold PBS.

Aliquot the cell batch into 1.0x10^7^cells/aliquot. At this point, the cell can be snapped in liquid nitrogen and stored -20^o^C for further biochemical experiments.

To start the IP assay from total cell lysate: We lyse the cells (one aliquot) in 300µl BC450 buffer (20mM Tris-HCl pH7.3, 10% glycerol, 450mM KCl, 0.1%NP40) for 30 minutes under shaking condition at 4^o^C.

Dilute the lysate by adding 600µl BC0 (20mM Tris-HCl pH7.3, 10% glycerol, 0.1%NP4) the total volume is increased up to 900 µl but salt concentration (KCl) decreases down to150mM. This diluted cellular lysate is the input for the IP assay.

We can start the IP by optionally reconstitute the nuclear extract into BC150 buffer (20mM Tris-HCl pH7.3, 10% glycerol, 0.1%NP40, 150mM KCl) as the input material for the IP assay.

Incubate the input amount correlating to 5x10^6^cells with 2.5-5µg antibody in the 1.5ml Eppendorf tube for 5hrs. In parallel, the control assay is carried out using equal amount of input material and equal amount of pre-immune serum.

Equilibrate protein G-beads by washing the G-beads with two times buffer BC150.

Precipitate the IP complexes by adding 30-40µl equilibrated protein G beads and extend the incubation time for 45 minutes more.

Centrifuge for 3 minutes at 3000xG to settle down protein G beads.

Wash the protein G beads with 800µl BC150 (20mM Tris-HCl pH7.3, 10% glycerol, 0.1%NP40, 150mM KCl) buffer by shaking on the Thermomixer comfort (Eppendorf, Hamburg, Germany) for 5 minutes at 8^o^C.

Repeat this washing step for two times more.

Elute the bound materials by heating up the beads at 95^o^C in 50µl 2xLaemmi SDS-PAGE sample loading buffer. At this point, the samples can be stored at -20^o^C for further analysis.

Note: The three buffers BC0, BC150, BC 450 are supplemented with fresh 1mM DTT, protease and phosphatese inhibitors and bensonase

The KCl concentration in the binding buffer and washing buffer is flexible depending on how strong the interaction of interest is

### Chromatin immuno-precipitation:

50ml aliquots of 5x10^5^cells/ml Molt4 cells is treated with 1% formaldehyde (final concentration) at RT for 5 minutes and then with 10mM Disuccinimidyl glutarate (final concentration) for another 2 minutes at RT.

The cross-linked cells are then spun down and washed 3 times with cold PBS.

Transfer the cells into 2.0 ml Eppendorf tube. PBS buffer then is completely removed.

Re-suspend the cells in 850µl cold-L1 lysis buffer; keep on ice for 10 minutes.

Spin the cells down at 800xG for 5 minutes.

Remove cytoplasm fraction (supernatant) and resuspend the pellet (cross-linked nuclei) in 850µl cold-L2 lysis buffer. At this point, the cross-linked nuclei can be snapped down in liquid nitrogen and stored at -80^o^C, otherwise we proceed to the next step.

Sonify the cross-linked nuclei by applying 7 times of 10 pulses of 50% amplitude on ice using Sonics-Vibra Cell-VCX 130PD Sonifier (Sonics& Materials Inc-Newtown CT, USA) in combination with CV188 ultra-sound converter and VC-502mm micro-tip 6300423. At this step, the cross-linked chromatin is expected to be sheared into 300-1000bp lengths.

The sheared chromatin amount corresponding to one aliquot of cross-linked nuclei (25x10^6^ cells) are then diluted 10 times into DB buffer and pre-cleared against 200µl-DB buffer-washed protein G slurry for 1hr.

To set up the immune-precipitation: 1.5 ml sheared, diluted chromatin (corresponding to 2.5-4x10^6^cells) is incubated with 2.5 to 5µg antibody for 5hrs. In parallel, the control assay is carried out using the same amount of input chromatin and equal amount of pre-immune serum.

Precipitate the DNA-protein by adding 30-40µl salmon sperm saturated protein G and extend the incubation time for 45 minutes more.

Centrifuge for 3 minutes at 10000xG to settle down protein G beads.

Remove thoroughly the supernatant and from now on, the samples (protein G beads) are kept away from any gemomic DNA containing solution.

Wash the protein G beads with CHIP-NaCl wash buffer by shaking on the Thermomixer comfort (Eppendorf, Hamburg, Germany) for 5 minutes at 8^o^C.

Repeat this washing step for two times more.

Optionally (depend how high antibody affinity is) one can proceed 1-3 washing steps more using CHIP-NaCl wash buffer.

Wash three time using TE buffer.

Release the bound materials by incubating protein G beads with 150µl 2% SDS in TE buffer for 5 minutes at 65^o^C.

Pool the two eluates from one immune-precipitation assay together and heat up the eluted sample at 70^o^C for at least 6hrs or even overnight.

Treat the sample with 2.5 µg protease K for 1hrs at 42^o^C.

Perform the DNA purification using Qiagen PCR cleaning kit.

Perform the qPCR to quantify the enrichment of suspect promoters (genomics region) in respect to control region.

Note:

The energy for chromatin shearing (pulse number, times, amplitude percentage) is defined empirically depending on individual cell lines.

Prior to start the IP assay chromatin shearing efficiency and antibody quality need to be tested: To test chromatin shearing efficiency we run a small amount of sonified chromatin sample against the 1% argarose gel with such relevant DNA marker and stain with ethilium brominde.

To test antibody affinity, we perform an IP assay using the sheared chromatin sample as the input, then pursuit immune-blot to detect the enrichment of the suspected protein.

All the buffers (L1, L2 DB) are added freshly with protease, phosphatese, inhibitors and histone deacetylase inhibitor (sodium butyrate).

The protein G beads used in the IP step are saturated with 1µg/ml sonified salmon sperm DNA and stored in DB buffer containing 0.02% NaN_3_

**Material needed**

**CHIP-lysis buffer L1:** 50mM Tris-HCl, pH 8.0, 2mM EDTA, 0.1% NP40, 10% Glycerol, 2mM fresh DTT, fresh protease inhibitors, fresh phosphatase inhibitors, 1mM sodium butyrate (Histon deacetylase inhibitor)

**CHIP-lysis buffer L2**: 50mM Tris-HCl, pH 8.0, 2mM EDTA, 1% SDS. 2mM fresh DTT, fresh protease inhibitors, fresh phosphatase inhibitors, sodium butyrate (Histon deacetylase inhibitor)

**CHIP-binding buffer**: 50mM Tris-HCl, pH 8, 5mM EDTA, 200mM NaCl, 0.5% NP40, 2mM fresh DTT, fresh protease inhibitors, fresh phosphatase inhibitors, sodium butyrate (Histon deacetylase inhibitor)

**CHIP-NaCl wash buffer:** 20mM Tris-HCl, pH 8, 2mM EDTA, 500mM NaCl, 1% NP40, 1%SDS, 2mM fresh DTT, fresh protease inhibitors, fresh phosphatase inhibitors, sodium butyrate (Histon deacetylase inhibitor)

**CHIP-LiCl wash buffer:** 20mM Tris-HCl, pH 8, 2mM EDTA, 500mM LiCl, 1% NP40, 1%SDS, 2mM fresh DTT, fresh protease inhibitors, fresh phosphatase inhibitors, sodium butyrate (Histon deacetylase inhibitor).

### Calcium-phosphate-mediated transfection of the packaging cell line

Plate Phoenix or Plat-E cells at about 20% confluency in 9 ml medium into a petri dish (Ø 10 cm) 24 hrs before transfection

Replace the medium 1hrs before transfection

Mix 20 µg DNA (retroviral bicistonic vector), 20µl TE buffer, 25 µl 2.5 M CaCl_2_ and water to a final volume of 250 µl

Ad 250 µl 2xHEBS Buffer, mix vigorously and incubate for 20 min at RT

Apply the mixture dropwise on the cells while gently shaking the dish

Incubate overnight and change medium

Harvest the supernatant containing the retrovirus on days 2 and 3 after transfection

### Transduction of mouse target cells with retrovirus containing supernatant

(Because the Plate Phoenix or Plat-E packaging cells in our lab are ecotropic, therefore the packed recombinant retrovirus released from them is only infect-able for mouse cells)

Plate out target cells in 12-well plate in a small volume of medium, and add undiluted cellulose acetate filtered retrovirus supernatant containing 4 µg/ml polybrene (hexadimethrine bromide); the final cell concentration should be about 3x10^5^ ml^-1^.

Spin the plate (1800 rpm, 34°C, and 45 minutes)

Incubate the plate in the incubator for at least six hrs

Remove some medium from on top of the cells, and repeat the infection with more fresh retroviral supernatant

If human cell line is the target then the recombinant retrovirus need to packed in an amphotropic cell lines or in our lab we use the mouse fibroblast derivative pantropic cells PT-67 (from Clontech) as the secondary packaging system for infecting human cell lines. That mean, recombinant retrovirus (supernatant) released from Phoenix or Plat-E cells is used for infecting PT-67 cells as described for mouse cell infection above.

Two days after infection, we use puromycin or fluorescent marker to select for virus producing PT-67 cells. From now on, any supernatant from the infected PT-67 cells is treated with care otherwise it may infect yourself.

An alternative way to make human infect-able virus producing cell lines is directly perform Calcium-phosphate-mediated transfection of puromycin resistant cassette integrated MSCV-based bicistronic plasmid onto PT-67 cell.

Two day after transfection, puromycin is used to select for stable virus producing PT-76 cells.

Once the virus containing supernatant is available, plate out human target cells in 12-well plate in a small volume of medium, and add undiluted cellulose acetate filtered retrovirus supernatant containing 4 µg/ml polybrene (hexadimethrine bromide); the final cell concentration should be about 3x10^5^ ml^-1^.

Spin the plate (1800 rpm, 34°C, and 45 minutes)

Incubate the plate in the incubator for at least six hrs

Remove some medium from on top of the cells, and repeat the infection with more fresh retroviral supernatant

Use puromycin or fluorescent marker to select for human cell lines expressing your cDNA of interest.

### Expression and Purification of GST tagged proteins

Insert DNA encoding for proteins of interest are cloned downstream in frame with Glutathione S Transferase (GST) coding sequence of pGEX-4T-1 vector and transform into E coli either (DN5α or BL21-CodonPlus).

The transformed bacteria harboring GST tagged protein coding sequence is pre-incubated at 37^o^C overnight then dilute with fresh LB medium down to OD ≈ 0.6. Reduce the culturing temperature to 18^o^C and continue grow for 15 minutes.

Induce the expression of recombinant protein by adding IPTG to final concentration between 0.05 -0.5mM. Maintain this culturing status for 2-4hs more, during this time the recombinant protein is expressing and accumulate into bacteria cells.

After 2-4h of expression, the bacteria is harvested by centrifuging down at 4000-5000xg. The supernatant is dumped out and at this point the bacteria pellet can be stored at -20^o^C or proceeded to the next step.

Put 4-6ml GST lysis buffer containing fresh DTT, phosphatase and protease inhibitors per one-gram bacteria pellet. Lyse the bacteria by sonification at 60% output for 50 strokes of 10 seconds using Sonifier Branson 450.

Pre-clear by centrifuging at higher than 30000g for 30 minutes, collect the supernatant.

Incubating the pre-clear lysate with Glutathion Sepharose TM 4 Fast Flow bead (the amount of bead is defined empirically depending on the level of expressed protein, for trial we use 1ml bead slurry/5ml lysate) for 1h at 4^o^C.

Wash the bead intensively for at least 3 times at 4^o^C, one can even perform the washing overnight. At this step, the recombinant proteins can be store as immobilized ligands in the GST wash buffer containing 0.02% sodium azide or elute with GST eluting buffer.

To elute the GST tagged protein, the bead is poured into gravity chromatography column, one wash more is required then elution is performed fractionally. For many biochemical experiments, removal of glutathione from eluate is required otherwise; the released protein can be stored at -80oC.

**Material needed:**

GST lysis or GST Wash buffer: 20mM Tris-HCl pH7.3 or 20mM Hepes-KCl, pH 7.9, 250mM NaCl, 10% Glycerol, 0.1% NP40, 1mM EDTA, fresh 1mDTT, fresh protease inhibitors, fresh phosphatase inhibitors,

GST eluting buffer: is GST Wash buffer containing 20mM Glutathione, 0.02% sodium azide

### GST pull-down assay:

The input materials for GST pull-down can be prepared as total cell lysate or nuclear extract but in both case it is necessary to reconstitute those inputs into the binding buffer BC150(20mM Tris-HCl pH7.3, 10% glycerol, 0.1%NP40, 150mM KCl, 2mM fresh DTT, fresh protease and phosphatase inhibitors).

The bait is GST-fused protein beads or GST- fused-mutated protein beads or just GST-beads are prepared as immobilized ligands (see “Expression and Purification of GST tagged proteins”)

Before performing GST pull-down assay, the quality of column materials needs to be tested to make sure that densities of immobilized ligands are equal between the real pull-down and mock control assay.

Wash to equilibrate the column materials two times with binding buffer BC150(20mM Tris-HCl pH7.3, 10% glycerol, 0.1%NP40, 150mM KCl, 2mM fresh DTT, fresh protease and phosphatase inhibitors).

Incubate the input amount correlating to 5x10^6^cells with 30µl equilibrated column material slurry (GST-fused-beads) in a 1.5ml Eppendorf tube for 5hrs. In parallel, the control assay is carried out using equal amount of equilibrated mock beads (GST only or mutated bait-GST beads) and equal amount of input.

Centrifuge for 3 minutes at 3000xG to settle down beads, remove the supernatant completely

Wash the beads with 800µl BC150 (20mM Tris-HCl pH7.3, 10% glycerol, 0.1%NP40, 150mM KCl) buffer by shaking on the Thermomixer comfort (Eppendorf, Hamburg, Germany) for 5 minutes at 4-8^o^C.

Repeat this washing step for two times more.

Elute the bound materials by heating up the beads at 95^o^C in 50µl 2xLaemmi SDS-PAGE sample loading buffer. At this point, the samples can be stored at -20^o^C for further analysis.

Material needed

GST pulldown buffer: 20mM Tris-HCl pH7.3 or 20mM Hepes-KCl, pH 7.9, 150mM KCl, 10% Glycerol, 0.05% NP40, 1mM EDTA, fresh 1mDTT, fresh protease inhibitors, fresh phosphatase inhibitors.

### Calcium phosphate transfection

Prepare the following solutions:

2X HEBS (HEPES Buffered Saline pH 7.05 +/-0.05: 280mMNaCl, 10mM KCl, 1.5mMNa_2_HPO_4_, 12mMDextrose, 50mM HEPES,) and 2.5M CaCl_2_ in 10mM HEPES

DEMEM/high glucose + 1% sodium pyruvate + 10% fetal calf serum + antibiotics

The following protocol is applicable for transfecting adherent human embryonic kidney 293 or mouse fibroblast NIH3T3 line and their derivatives.

One day prior to transfection, the cells should be split into 25-30% confluent so that they are logarithmically growing on the day of transfection, in our experiment the 293-cell culture was 50% confluent at the time of transfection.

Prepare 1µg transfecting DNA mixture per 4cm^2^ of cell-adhered surface.

Change the medium one hour before transfection.

Mix 20 µg DNA with 20µl TE buffer, 25 µl 2.5 M CaCl_2_ and water to a final volume of 250 µl

Ad 250 µl 2xHEBS Buffer, mix vigorously and incubate for 20 min at RT

Incubate at room temperature for 25 minutes to form DNA/calcium precipitates.

Apply the DNA/calcium precipitates dropwise to the surface of the media containing the cells. Swirl the plate gently to mix.

After overnight incubation, remove the calcium phosphate containing medium. Replace to with normal medium.

Enzymatic assays of expressed genes in transfected cells are performed 48-72hrs post transfection.

### Beta galactosidase assay

Prepare ONPG Beta gal buffer:1.1mM MgCl_2_, 82mM Na_2_HPO_4_, 18mM NaH_2_PO_4_ freshly 1mg/ml 2-Nitrophenyl β-D Galactopyrannoside-Sigma, 50mM fresh β mecaptoethanol.

Transfer 200µl ONPG Beta gal buffer into Nunclone Surface 96 well plate.

Add 20µl cell lysate to each well and incubate at 37^o^C for 15 minutes

Measure the end-point absorbance at 410nm wavelength in microplate reader Molecular Devices Spectra Max 250.

### Luciferase reporter assay

Harvest the 293 cells just by using the 10ml stripette to intensively suck up and down but in case of working with other stronger adherent cell like NIH3T3, Hela, or Min6… one would have to use the scrapper to strip off the cell.

Wash the cell two times by cold PBS.

Lyse the cell in Pomega Reporter Lysis Buffer (we use 300µl for 1.107Cells); keep this suspension on ice for 15 minutes to help the cell to be completely lysed.

Spin at 10,000 rpm at 40C for 10 minutes to pellet cell debris, the supernatant cell lysate is collected and can be frozen at -20^o^C for Luciferase activity assay.

Transfer 20µl, 10 µl, 5µl cell lysate into luminometer tubes 5ml, 75x12mm Φ (Sarstedt, Germany). Tap the side of the tubes containing the lysate to gently mix the contents. Place a tube in the Lumat LB 9507 luminometer (Berthold technologies GmbH, Bad Wildbad, Germany).

To start the assay, inject 100 µl of Pomega luciferin substrate solution into the luminometer tube and measure the light output at 562nm for a period of 2-60 seconds at room temperature.
